# Accelerated Mechanophore Activation and Drug Release in Network Core‐Structured Star Polymers Using High‐Intensity Focused Ultrasound

**DOI:** 10.1002/smsc.202400082

**Published:** 2024-06-17

**Authors:** Jilin Fan, Mingjun Xuan, Kuan Zhang, Rostislav Vinokur, Lifei Zheng, Robert Göstl, Andreas Herrmann

**Affiliations:** ^1^ Institute of Technical and Macromolecular Chemistry RWTH Aachen University Worringerweg 2 52074 Aachen Germany; ^2^ DWI – Leibniz Institute for Interactive Materials Forckenbeckstr. 50 52056 Aachen Germany; ^3^ Wenzhou Institute University of Chinese Academy of Sciences Jinlian Road 1 Wenzhou 325001 China; ^4^ Department of Chemistry and Biology University of Wuppertal Gaußstr. 20 42119 Wuppertal Germany

**Keywords:** drug release, mechanophores, polymer mechanochemistry, sonopharmacology, ultrasound

## Abstract

The ultrasound (US)‐induced activation of mechanophores embedded in linear polymers (LPs) is the most widely employed technique to realize chemical function by polymer mechanochemistry. However, the commonly used US frequency in this context is around 20 kHz, producing strong inertial cavitation limiting biomedical applicability. Herein, 20 kHz US and 1.5 MHz high‐intensity focused US (HIFU) are investigated to drive disulfide mechanophore activation and mechanochemical polymer chain scission in network core‐structured star polymers (NCSPs). It is found that the efficiency of activating disulfide mechanophores in NCSPs using 1.5 MHz HIFU irradiation is similar to the efficiency achieved with 20 kHz sonication. This is quantified by ‘turn on’ sensor molecules leveraging the Michael addition of the mechanochemically generated thiol groups and subsequent retro Diels–Alder reaction to release a fluorophore. Moreover, the anticancer drug doxorubicin (Dox) covalently loaded into NCSPs is efficiently released by 1.5 MHz HIFU. Finally, an in vitro study of drug release from NCSPs is performed, demonstrating the potential of HIFU‐activated polymer mechanochemistry for sonopharmacology.

## Graphical abstract

Contrary to linear polymers, the efficiency of mechanophore chain scission in network core‐structured star polymers with 1.5 MHz HIFU irradiation is comparable to the efficiency achieved with 20 kHz sonication. Although the spanning molar masses of star and linear polymers are comparable, a 108 times faster mechanochemical bond scission than linear polymers under HIFU irradiation is achieved.

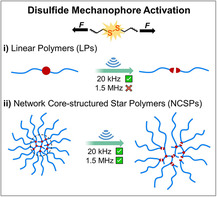

## 1 Introduction

Ultrasound (US)‐induced polymer mechanochemistry has gained significant attention for applications in biological systems, such as for the activation of drugs (sonopharmacology),^[^ ^1^ ^]^ proteins,^[^ ^2^ ^]^ and biomaterials.^[^ ^3^ ^]^ US exhibits several advantages over light,^[^ ^4^ ^]^ heat,^[^ ^5^ ^]^ pH,^[^ ^6^ ^]^ or redox potential,^[^ ^7^ ^]^ for example, widespread clinical use, high tissue penetration depth, and precise spatiotemporal control. Yet, the commonly applied frequency of US in current polymer mechanochemical systems is 20 kHz.^[^ ^1^, ^2^, ^3^, ^8^ ^]^ This frequency generates strong inertial cavitation, which is destructive to tissues and causes damage to the biological system.^[^ ^9^ ^]^

Conversely, high‐intensity focused ultrasound (HIFU) driven in the MHz frequency range is an established modality in biomedical applications^[^ ^10^ ^]^ including tumor ablation,^[^ ^11^ ^]^ pain management,^[^ ^12^ ^]^ neurosurgery,^[^ ^13^ ^]^ and drug release.^[^ ^14^ ^]^ A metric to quantify the biosafety of such high‐frequency US is the mechanical index (MI) where an MI < 1.9 is considered medically safe as approved by the FDA.^[^ ^15^ ^]^ HIFU is, however, often operated above this safety limit for therapeutic applications that exploit destructive mechanical properties and thermal effects of sonication, such as cancer treatment.^[^ ^16^ ^]^ Consequently, there is a fundamental demand to develop polymer mechanochemical reactions that can be driven at medically tolerable US intensities consolidating the mechanochemical principles with the biomedical framework conditions.

Recently, Li, Moore, and coworkers reported the in vitro mechanochemical free radical generation in hydrogels for noninvasive cancer therapy.^[^ ^1e^ ^]^ They activated azo mechanophores by HIFU with spatiotemporal precision to generate free radicals that converted to reactive oxygen species for the inhibition of cancer cell growth. Robb, Shapiro, and co‐workers reported a synergistic platform that coupled the selective mechanophore activation in linear polymers (LPs) with biocompatible focused US by leveraging pressure‐sensitive gas vesicles as acousto‐mechanical transducers.^[^ ^1f^ ^]^ While the use of auxiliaries, such as microbubbles,^[^ ^17^ ^]^ hence can increase the sensitivity of conventional polymers to US thereby opening up a pathway to biomedical applications, another viable approach is the modification of the polymer architecture itself in which the mechanophores are localized.^[^ ^18^ ^]^ The acceleration of mechanochemical bond scission has thereby been demonstrated in dendrimers, polymer brushes, and microgels.^[^ ^19^ ^]^

Star polymers are another polymer architecture investigated in the context of polymer mechanochemistry and their respective mechanophore activation efficiencies as compared to linear chains have been studied.^[^ ^20^ ^]^ These investigations confirm a mechanistic interpretation of star polymer chain scission that is governed by the spanning rather than total molar mass. Yet, these works exclusively focus on three‐armed star polymers where only one mechanophore is located near the junction point. However, star architectures are far more diverse and, for example, network core‐structured star polymers (NCSPs) with a potential multimechanophore core structure and multiple long end chains can be conceived. Yet, the mechanochemical activity of such NCSPs has not been investigated previously, although a systematic study would further increase our understanding of chain scission of star polymers, improve predictive capabilities for mechanophore activation, and inform rational polymer design.

**Scheme 1:**
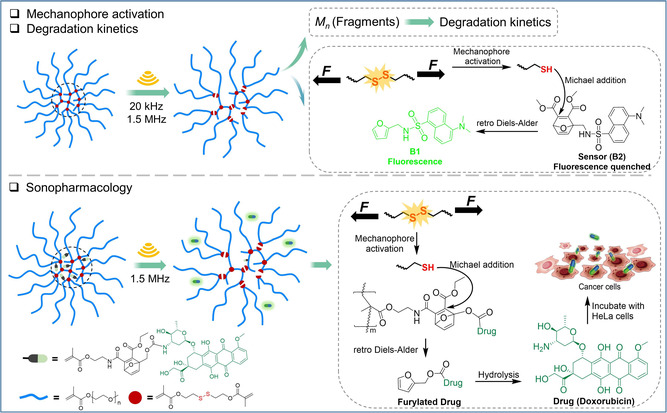
Schematic illustration of the mechanochemical NCSP fragmentation, activation of disulfide mechanophores, and drug release upon US irradiation.

Here we prepare NCSPs containing multiple disulfide mechanophores by reversible addition fragmentation chain‐transfer (RAFT) polymerization (**Scheme** **1**). Both 20 kHz US and 1.5 MHz HIFU are applied to verify the mechanochemical responsivity of NCSPs. We quantitatively assess disulfide mechanophore activation and the chain fragmentation of NCSPs. Importantly, mechanophore‐free network core‐structured star polymers (FNCSPs) as control material indicate the significant influence of the mechanophores on overall mechanochemical bond scission. The superior mechanophore activation of NCSPs is underscored by comparison of the scission kinetics to LPs. Based on these results, we demonstrate a proof‐of‐concept drug release using 1.5 MHz HIFU with an MI of 2.4. Exposing HeLa cells to the released drug in vitro showcases the potential of mechanochemical drug release from NCSPs for sonopharmacology.

## 2 Results and Discussions

### 2.1 NCSP Preparation and Disulfide Mechanophore Activation

**Figure 1:**
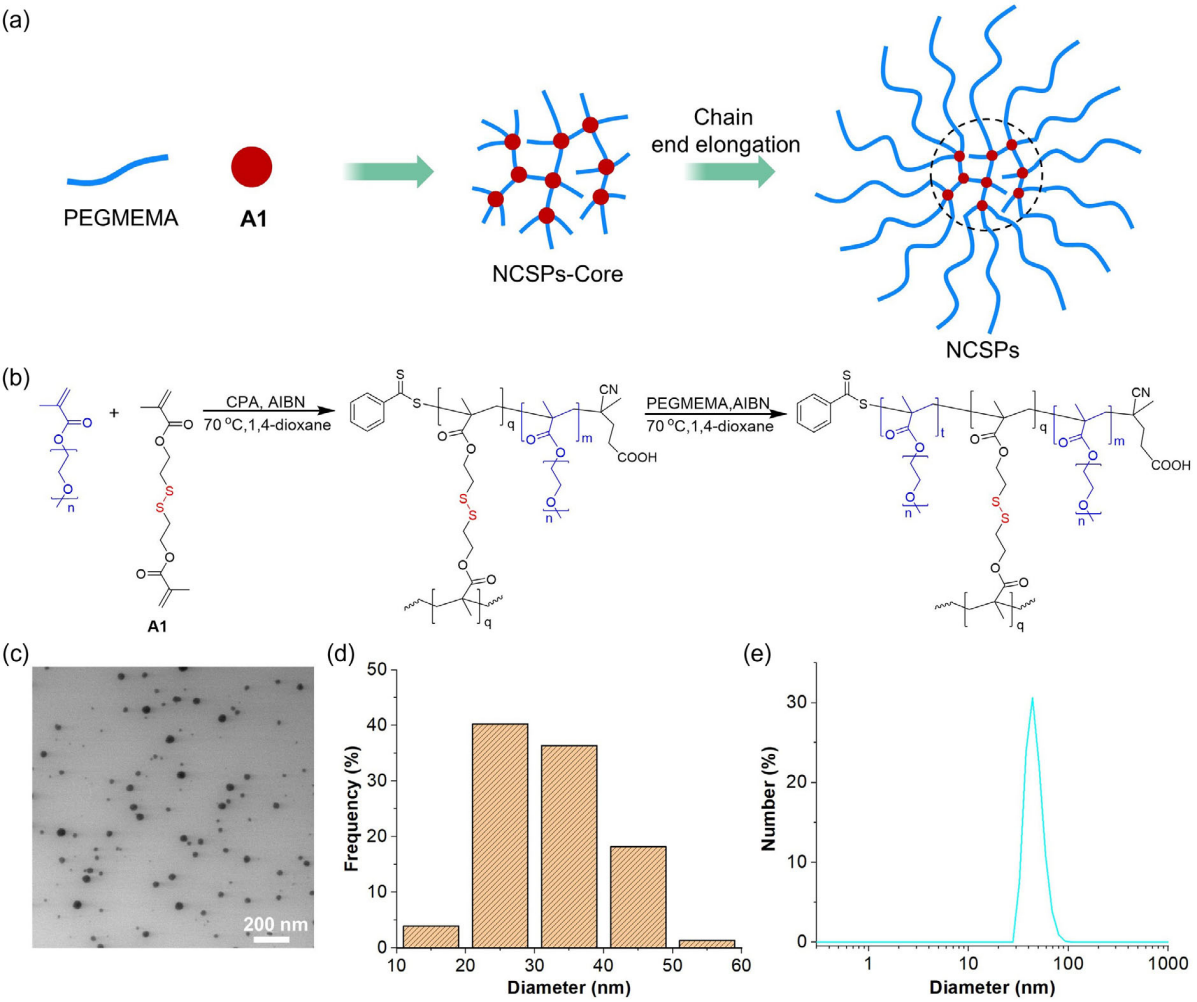
Synthesis and characterization of NCSPs. a) Schematic representation and b) chemical components of the synthesis process. Red: disulfide mechanophore; blue: PEGMEMA chains. c) TEM micrograph. d) Size distribution histogram compiled from panel c). e) DLS.

The disulfide crosslinker **A1** for NCSP preparation was synthesized by methacryloylation of commercial 2,2′‐dithiodiethanol. From there, NCSPs were synthesized by RAFT copolymerization and chain extension from the endpoints of the NCSP core with the biocompatible PEG‐analogue poly(ethylene glycol) methyl ether methacrylate (PEGMEMA). The synthesis involved two steps: 1) the fabrication of the polymeric core architecture and 2) the growth of PEGMEMA chains on the surface of the polymeric core (**Figure** **1**a,b, and Scheme S3, Supporting Information). The molar mass of the NCSPs was very roughly ≈109 kDa measured by gel permeation chromatography (GPC) using a refractive index detector (Figure S7, Supporting Information). The morphology and size distribution of the NCSPs were studied using transmission electron microscopy (TEM, Figure 1c) and dynamic light scattering (DLS), respectively. The diameter of NCSPs was in the range of 20–50 nm (Figure 1d,e). In addition, the structure of the NCSPs was investigated by GPC and ^1^H NMR, the number of arms and degree of polymerization *X*_n_ of the arms were ≈7 and 37, respectively (see Supporting Information).

To assess the mechanophore activation in NCSPs, nonfluorescent 7‐oxanorbornadiene sensor **B2** containing latent fluorophore **B1** was synthesized alongside (Scheme 1, S1, Supporting Information).^[^ ^21^ ^]^ **B1** acted as a reporter, allowing the facile quantification of free thiols that reacted with **B2** in a Michael addition, whereupon fluorescent **B1** was released by the retro Diels–Alder reaction.

As kinetic control material, LPs with chain‐centered disulfide mechanophores were synthesized by Cu^0^‐mediated controlled radical polymerization (Scheme S2, Supporting Information). The chain growth process was controlled through the reaction time at room temperature. LPs were obtained after 6 h with *M*_n_ = 47 kDa and *Ð*_M_ = 1.4. These were subjected to sonication in H_2_O/DMSO (v:v, 4:1) in the presence of sensor **B2**, and aliquots were analyzed by GPC as well as fluorescence spectroscopy. Expectedly, significant fluorescence was recorded after using 20 kHz US at a sound intensity *I* of 12.4 W cm^−2^ (Figure S3 and S4, Supporting Information). Thereafter, we identified the intensity threshold of mechanophore activation and polymer chain scission. We found that no chain scission occurred below *I* = 1.5 W cm^−2^ using 20 kHz US (Figure S5, Supporting Information) and for all tested conditions using 1.5 MHz HIFU (Figure S6, Supporting Information).

**Figure 2:**
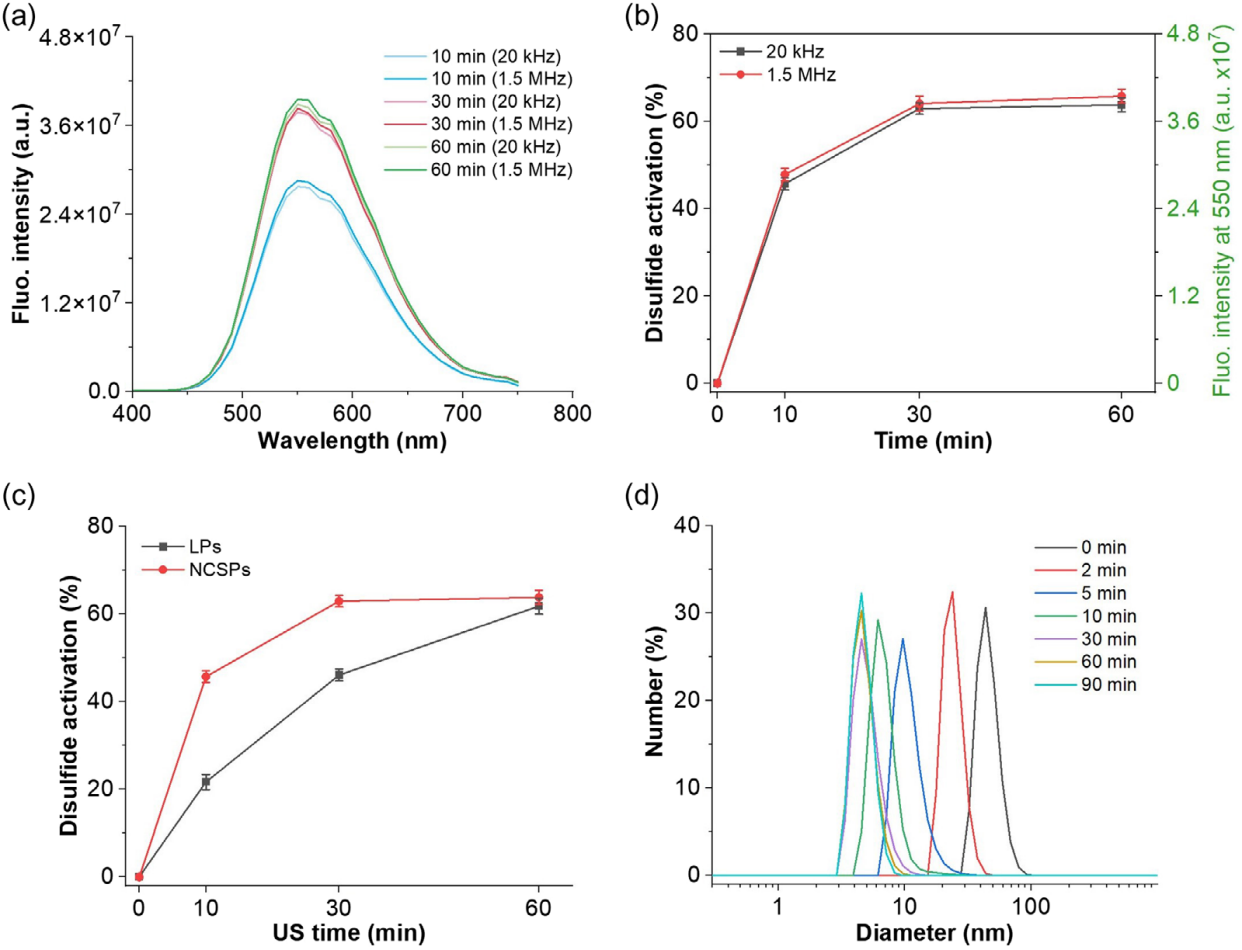
US‐induced disulfide mechanophore activation in NCSPs. a) Fluorescence spectra of the mixture of **B2** and NCSPs after 20 kHz (*I* = 12.4 W cm^−2^) and 1.5 MHz (*I* = 2300 W cm^−2^) sonication (*λ*_exc_ = 330 nm). b) Disulfide mechanophore activation after 20 kHz and 1.5 MHz sonication. Mean ± Standard deviation (SD) from the mean. *N* = 3 independent sonications. The data correspond to both *y*‐axes. c) Fraction of activated disulfide mechanophores in NCSPs compared to LPs using 20 kHz US at *I* = 12.4 W cm^−2^. Mean ± SD from the mean. *N* = 3 independent sonications. d) DLS of NCSP solutions over the course of their sonication using 20 kHz US at *I* = 12.4 W cm^−2^.

We then explored the activation of disulfide mechanophores in NCSPs through US. Upon sonication (20 kHz with *I* = 12.4 W cm^−2^ or 1.5 MHz with *I* = 2300 W cm^−2^, pulse sequence 2 s on, 1 s off) for 30 min, the green fluorescence of released **B1** was observed (Figure S12, Supporting Information). The fluorescence intensity was measured to quantify mechanophore activation after the respective 20 kHz and 1.5 MHz sonications (**Figure** **2**a). Samples treated by HIFU irradiation showed comparable fluorescence intensities to 20 kHz US in the same time regime under the chosen conditions (Figure 2b). The activated mechanophore fraction was calculated using the fluorescence of the mixture of **B2** and NCSPs treated with large excess of reducing agent tris(2‐carboxyethyl)phosphine (TCEP) as a maximum achievable value. More than 60% of **B2** were activated within 30 min at 20 kHz US using NCSPs, which was higher compared to LPs (Figure 2c). We attributed this difference to the higher molar mass and hence higher mechanochemical reactivity of the NCSPs.^[^ ^22^ ^]^ Additionally, the size distributions of NCSPs were measured by DLS. The hydrodynamic size considerably decreased within 10 min and dropped from around 50 nm to less than 10 nm after 30 min sonication underlining the occurring chain fragmentation (Figure 2d).

### 2.2 Mechanochemical Reactivity of NCSPs

**Figure 3:**
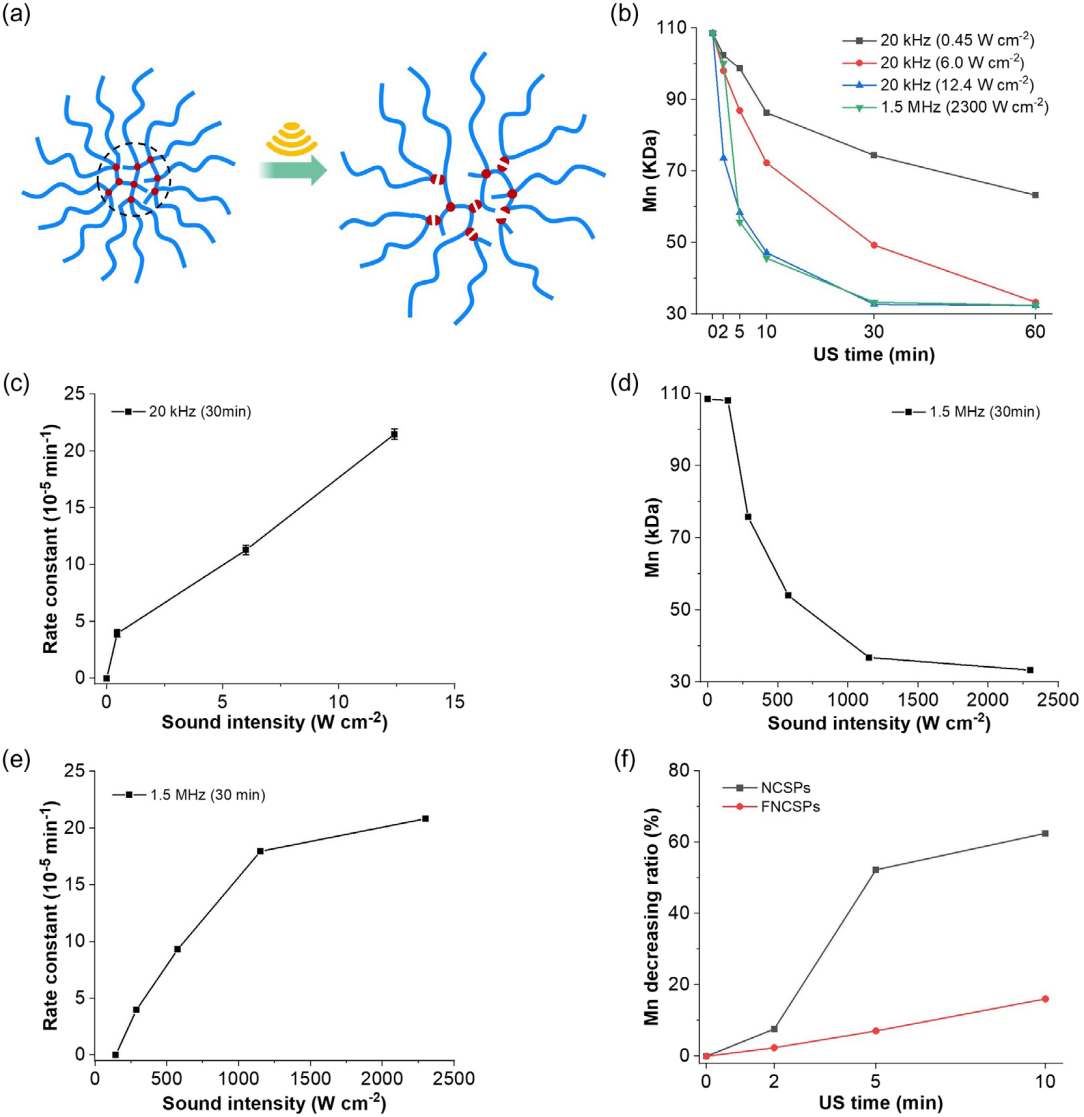
Mechanochemical reactivity analysis of NCSPs. a) Schematic representation of the degradation. b) *M*_n_ over the course of 20 kHz and HIFU sonication. c) *k*_app_ under 20 kHz sonication as a function of *I*. Mean ± SD from the mean. *N* = 3 independent sonications. d) Final *M*_n_ after 30 min sonication with HIFU at 1.5 MHz as a function of *I*. e) *k*_app_ under HIFU at 1.5 MHz sonication as a function of *I*. f) *M*_n_ decreasing ratio over the course of the sonication of NCSPs and FNCSPs with HIFU (*I* = 2300 W cm^−2^).

Hereafter, we systematically investigated the mechanochemical reactivity of the NCSPs depending on employed US frequency and *I* (**Figure** **3**). The applied *I* for 20 kHz and 1.5 MHz US are listed in Table S1 and S2, Supporting Information. In addition, we recorded the superposition of nonselective and selective chain scission as change in *M*_n_ (Table S4, Supporting Information) as a function of time (Figure 3b). Comparing the *M*_n_ before and after sonication revealed that the NCSPs decomposed rapidly within 30 min, and the *M*_n_ decreased to less than 30% of its initial value (Figure S16, Supporting Information). NCSP degradation commenced with a lower threshold *I* of 0.45 W cm^−2^ at 20 kHz compared to LPs where the threshold *I* was 1.5 W cm^−2^ underpinning the increased mechanochemical reactivity of the NCSPs toward inertial cavitation. At an *I* of 12.4 W cm^−2^, the NCSPs degraded completely within 30 min reaching the limiting molar mass. This was underscored by DLS measurements, which revealed no further reduction in hydrodynamic size after this time. In contrast, the ultimate *M*_n_ of LPs was only reached after 60 min under identical conditions.

The mechanochemical reactivity of the NCSPs was quantitatively assessed under 20 kHz sonication using a previously reported method^[^ ^23^ ^]^ by calculating the apparent scission rate constant *k*_app_. Expectedly, increasing *I* led to higher *k*_app_ (Figure 3c). Comparable observations were made for HIFU sonication at 1.5 MHz. While the threshold *I* for chain scission was determined to 290 W cm^−2^ and a corresponding MI of 2.4 (Figure 3d, Table S5, Supporting Information), *k*_app_ expectedly also increased with increasing *I* (Figure 3e).

The role of the disulfide mechanophores was then explored in comparison to mechanophore‐free, but similarly sized FNCSPs (Figure S17, Supporting Information). Over the course of 10 min HIFU sonication it became clear that the *M*_n_ of the FNCSPs decreased considerably slower compared to the NCSPs (Table S6, Supporting Information, Figure 3f) underscoring the significant contribution of the disulfide mechanophores to *k*_app_.

From previous reports, the concept of the spanning molar mass (*M*_span_) emerged.^[^ ^18^, ^20^ ^]^ These studies confirmed a mechanistic interpretation of star polymer chain scission that is governed by the spanning rather than total molar mass. To verify whether the reported conclusions were applicable to NCSPs as well, *M*_span_ of NCSPs and LPs were calculated (for details, see Supporting Information). NCSP star polymers with *M*_span_ = 54 kDa and *k*_app_ = 10.8 × 10^−5^ min^−1^ showed comparable performance to LPs with *M*_span_ = 47 kDa and *k*_app_ = 10.4 × 10^−5^ min^−1^ upon 60 min sonication at 20 kHz. The spanning molar mass concept thus holds true for LPs and NCSPs at 20 kHz sonication. However, under 1.5 MHz, HIFU irradiation at *I* = 2900 W cm^−2^ for 60 min NCSPs and LPs showed *k*_app_ of 10.8 × 10^−5^ and 0.1 × 10^−5^ min^−1^ (Table S8, Supporting Information) and thus an obvious rate difference in favor of the NCSPs. Therefore, we concluded that NCSPs showed a higher sensitivity to 1.5 MHz HIFU irradiation compared to LPs in chain scission or disulfide mechanophore activation.

### 2.3 Ultrasound‐Induced Drug Release from NCSPs

Having successfully shown the superior disulfide mechanophore activation in NCSPs by HIFU, we aimed to demonstrate the release of a furylated drug molecule in a proof‐of‐concept application. Therefore, we employed furylated Dox **D1**, which has previously shown promising anticancer activity in soft tissue, bone sarcomas, cancers of the breast, ovary, bladder, and thyroid.^[^ ^24^ ^]^ However, it exhibits a short half‐life as well as systemic and specific cardiac toxicity,^[^ ^25^ ^]^ rendering it an ideal candidate for remote‐controlled release.

**Figure 4:**
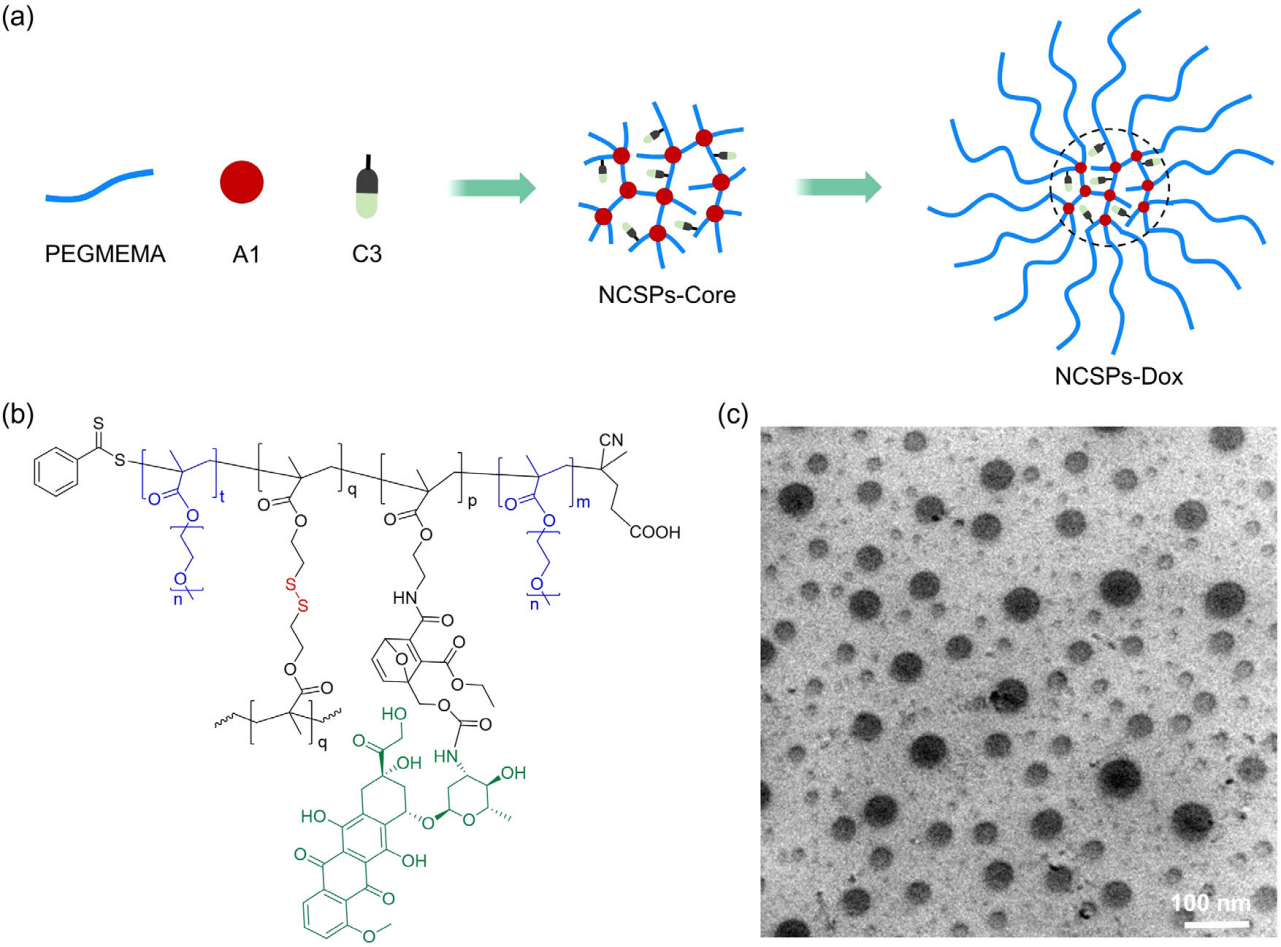
The synthesis of Dox‐bearing NCSPs. a) Schematic representation of the synthesis process and b) final polymer. Disulfide mechanophore, Dox, and PEGMEMA chains are highlighted in red, green, and blue respectively. c) TEM micrograph.

The acrylate‐bearing Diels–Alder adduct of furylated Dox **C3** was synthesized (**Figure** **4**a) and covalently incorporated into the NCSP core through copolymerization (Figure 4b). The morphology of the resulting NCSPs was analyzed using TEM (Figure 4c).

**Figure 5:**
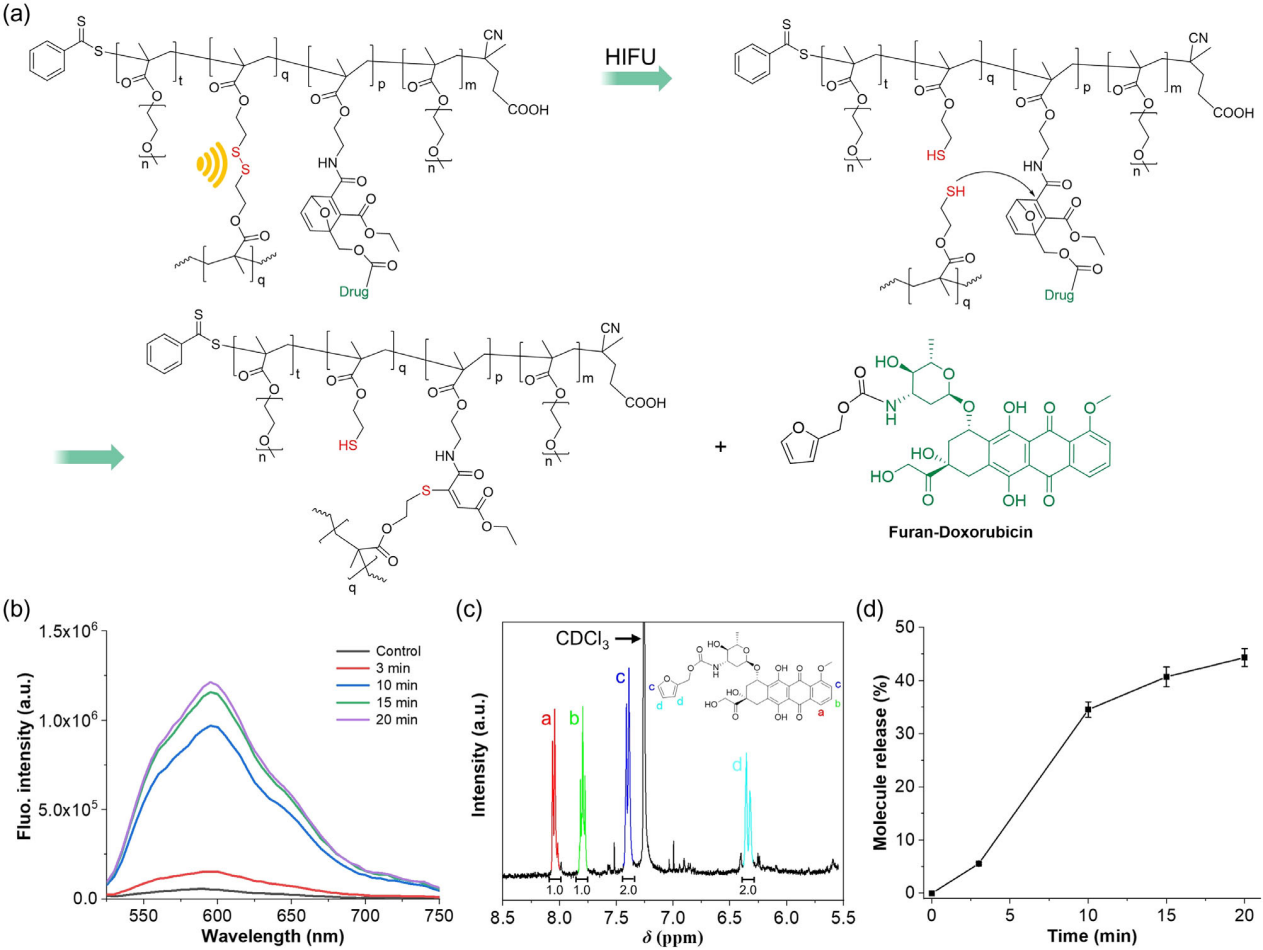
HIFU‐induced disulfide mechanophore activation and **D1** release from NCSPs. a) Schematic illustration. b) Fluorescence spectra (*λ*_exc_ = 485 nm) over the course of HIFU application (1.5 MHz, *I* = 2300 W cm^−2^). c) Section of ^1^H NMR (400 MHz, CDCl_3_) spectrum showing characteristic **D1** peaks. d) The release profile of **D1** from NCSPs over sonication time (1.5 MHz, *I* = 2300 W cm^−2^). Mean ± SD from the mean. *N* = 3 independent sonications.

HIFU at 1.5 MHz and *I* = 2300 W cm^−2^ was then used to release **D1** (**Figure** **5**a). Therefore, Dox‐loaded NCSPs were dispersed in a solvent mixture of DMSO and PBS (v/v = 1:4) and then sonicated for up to 20 min. Fluorescence spectra were collected at each time point to assess the release of fluorescent **D1** (Figure 5b). To further confirm these results, ultrahigh‐performance liquid chromatography–mass spectrometry (UPLC‐MS) analysis was conducted over the course of the sonication. Additionally underpinning the successful release, ^1^H NMR analysis demonstrated the emergence of characteristic peaks of **D1** in the aromatic region (Figure 5c).

To quantify released **D1**, a correlation between its concentration and fluorescence intensity was established (Figure S21, Supporting Information). To obtain a maximum possible value, 2‐mercaptoethanol (MCE) was used to reduce all disulfide bonds within the NCSPs (Figure S22a, Supporting Information). Thereby, a maximum release of **D1** by HIFU of around 40% was observed (Figure 5d). In addition, we performed the experiments in 10% DMSO, 5% DMSO, and pure water to explore the influence of solvent composition on US responsivity and molecular release. We found that with 5% DMSO, ≈20% of furylated Dox was released after 20 min HIFU irradiation. When the experiments were performed in water, ≈5% furylated Dox were released. (Figure S22c and S22e, Supporting Information).

### 2.4 In Vitro Cancer Therapy of Released Dox

**Figure 6:**
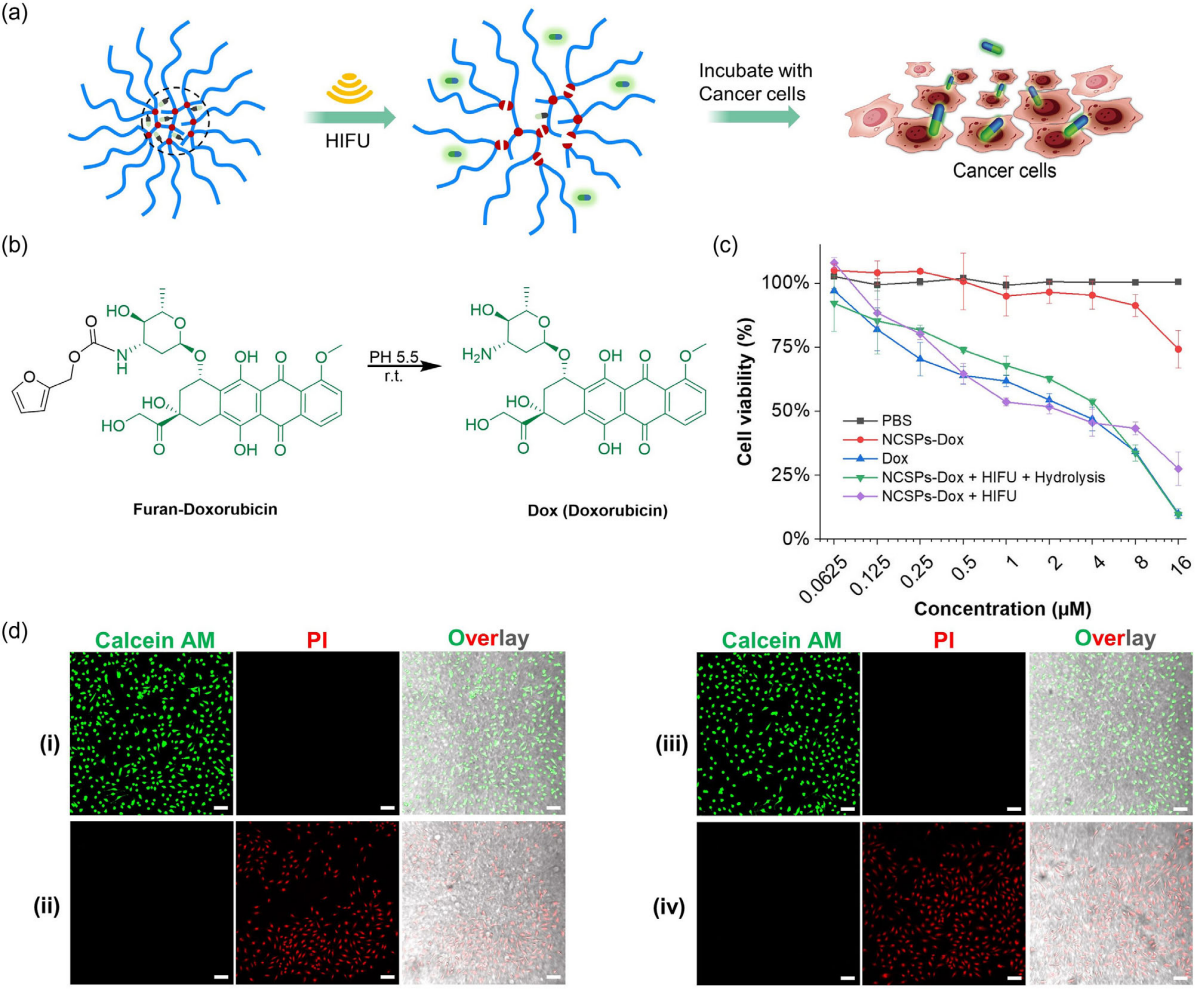
Dox‐loaded NCSPs for in vitro sonopharmacology. a) Schematic illustration of the drug release. b) Hydrolysis of released furylated Dox. c) Cell viability using MTS proliferation assay. Samples: PBS, Dox‐loaded NCSPs, Dox, Dox‐loaded NCSPs after HIFU and hydrolysis, and Dox‐loaded NCSPs after HIFU all ex situ mixed with HeLa cells. Mean values ± SD from the mean. *N = *3 independent sonications. d) CLSM micrographs of HeLa cells treated with different samples (live cells: calcein AM, green; dead cells: propidium iodide, red). i) PBS, ii) pristine Dox, iii) nonsonicated Dox‐loaded NCSPs, iv) sonicated and hydrolyzed Dox‐loaded NCSPs (1.5 MHz, *I* = 290 W cm^−2^, 30 min). HeLa cells were stained for 5 min. Scale bars: 50 μm.

Hereafter, the potential of Dox‐loaded NCSPs for in vitro treatment was investigated by MTS cell proliferation assay and confocal laser scanning microscopy (CLSM) assessing the performance of the delivered drug in suppressing cancer cell growth (**Figure** **6**a). The half‐maximal inhibitory concentrations (IC_50_) of PBS, Dox‐loaded NCSPs, pristine Dox, and Dox‐loaded NCSPs after HIFU treatment and hydrolysis were investigated. The hydrolysis process to defurylate released Dox was carried out in a solvent mixture of DMSO and PBS (v/v = 1:4) at a tumor‐like pH of 5.5 for 48 h (Figure 6b) and confirmed by UPLC (Figure S23, Supporting Information).^[^ ^26^ ^]^ Notably, the IC_50_ of the sonicated and defurylated Dox‐loaded NCSPs was significantly lower compared to the control samples and similar to pristine Dox (Figure 6c). While PBS expectedly was almost not cytotoxic under the applied conditions, HeLa cells treated with different concentrations of nonsonicated Dox‐loaded NCSPs still exhibited a high viability, indicating that the intact drug carrier is relatively biocompatible. We attributed this to the hydrodynamic screening of the Dox‐loaded cores by the solvated PEGMEMA chains. In addition, the sonicated Dox‐loaded NCSPs before hydrolysis were investigated and revealed that furylated Dox also has an inhibitory effect on HeLa cell growth. This could either be due to the in situ defurylation in the cancer cells or due to the intrinsic cytotoxicity of furylated Dox. Furthermore, CLSM was performed to underpin these results after live cell staining with calcein AM and dead cell staining with propidium iodide. The number of live HeLa cells significantly decreased by treatment with pristine Dox or sonicated and hydrolyzed Dox‐loaded NCSPs (Figure 6c) underlining the success of HIFU‐induced disulfide mechanophores activation and the subsequent Dox release from NCSPs for sonopharmacology.

## 3 Conclusion

We here showed the accelerated US‐induced disulfide mechanophore activation in NCSPs that showed a higher sensitivity to US compared to LPs. We quantified the mechanophore activation by Michael addition of the mechanochemically generated thiols to a 7‐oxanorbornadiene scaffold carrying the latent fluorescent cargo, which was then released by retro Diels–Alder reaction. We found that NCSPs enabled faster mechanophore scission and were responsive to lower sonication powers compared to linear chains bearing an identical mechanophore in their center. Moreover, we systematically investigated how mechanochemical NCSP fragmentation depended on the sonication conditions in terms of frequency and *I*. While linear chains were only responsive to 20 kHz US, which produces strong inertial cavitation, NCSPs were also activated with biomedically used 1.5 MHz HIFU. These findings underline the critical role of polymer architecture for the efficient activation of mechanophores.^[^ ^18^ ^]^ We then used this system for a proof‐of‐concept drug release experiment and liberated Dox by 1.5 MHz HIFU with an MI of 2.4 to inhibit the growth of cancer cells in vitro. These findings constitute an important step on the way to consolidate polymer mechanochemistry with biomedically tolerable ultrasonication conditions.

## Conflict of Interest

The authors declare no conflict of interest.

## Supporting information

Supplementary Material

## Data Availability

The data that support the findings of this study are openly available in Zenodo at https://zenodo.org/records/11240252.
