# Supplementary material for: Accelerated Mechanophore Activation and Drug Release in Network Core‐Structured Star Polymers Using High‐Intensity Focused Ultrasound
Source: Small Sci. 2024 Jun 17;4(8):2400082. doi: 10.1002/smsc.202400082 (PMC11935275; doi:10.1002/smsc.202400082)
Supplement: Supplementary file 1 — Supplementary Material [file SMSC-4-2400082-s001.pdf]

Supporting Information

for

Accelerated Mechanophore Activation and Drug Release in Network Core-Structured Star Polymers Using High-Intensity Focused Ultrasound

Jilin Fan, Mingjun Xuan, Kuan Zhang, Rostislav Vinokur, Lifei Zheng, Robert Göstl, and Andreas Herrmann**

J. Fan, M. Xuan, K. Zhang, R. Göstl, A. Herrmann
Institute of Technical and Macromolecular Chemistry, RWTH Aachen University, Worringerweg 2, 52074, Aachen, Germany
E-mail: xuan@dwi.rwth-aachen.de; herrmann@dwi.rwth-aachen.de

J. Fan, M. Xuan, K. Zhang, R. Vinokur, R. Göstl, A. Herrmann
DWI – Leibniz Institute for Interactive Materials, Forckenbeckstr. 50, 52056 Aachen, Germany

K. Zhang, L. Zheng
Wenzhou Institute, University of Chinese Academy of Sciences, Jinlian Road 1, 325001 Wenzhou, China

R. Göstl
Department of Chemistry and Biology, University of Wuppertal, Gaußstr. 20, 42119 Wuppertal, Germany

Content

1. Experimental Section ... 3

1.1. Materials ... 3

1.2. Methods ... 3

1.2.1. General Instrumentation ... 3

1.2.2. Fluorescence Spectroscopy ... 4

1.2.3. Sonication Experiments ... 4

1.2.4. Cell Imaging ... 4

1.2.5. MTS Proliferation Assays ... 4

1.2.6. Quantification Method of Disulfide Activation Percentage ... 5

2. Synthetic Procedures and Characterization Data ... 6

2.1. Disulfanediylbis(ethane-2,1-diyl) bis(2-methylacrylate) (**A1**) ... 7

2.2. 5-(dimethylamino)-N-(furan-2-ylmethyl)naphthalene-1-sulfonamide (**B1**) ... 9

2.3. Dimethyl 1-(((5-(dimethylamino)naphthalene)-1-sulfonamido)methyl)-7-oxabicyclo[2.2.1]hepta-2,5-diene-2,3-dicarboxylate (**B2**) ... 11

2.4. Ethyl 4-((2-(methacryloyloxy)ethyl)amino)-4-oxobut-2-ynoate (**C1**) ... 13

2.5. Ethyl 1-(hydroxymethyl)-3-((2-(methacryloyloxy)ethyl)carbamoyl)-7-oxabicyclo[2.2.1]hepta-2,5-diene-2-carboxylate (**C2**) ... 15

2.6. Ethyl 1-(((((2S,3R,4S)-3-hydroxy-2-methyl-6-(((1S,3S)-3,5,12-trihydroxy-3-(2-hydroxyacetyl)-10-methoxy-6,11 -dioxo-1,2,3,4,6,11-hexahydrotetracen-1-yl)oxy)tetrahydro-2H-pyran-4-yl)carbamoyl)oxy)methyl)-3-((2 (methacryloyloxy)ethyl)carbamoyl)-7-oxabicyclo[2.2.1]hepta-2,5-diene-2-carboxylate (**C3**) ... 17

2.7. Furan-2-ylmethyl ((2S,3R,4S,6R)-3-hydroxy-2-methyl-6-(((1S,3S)-3,5,12-trihydroxy-3-(2-hydroxyacetyl)-10-methoxy-6,11-dioxo-1,2,3,4,6,11-hexahydrotetracen-1-yl)oxy)tetrahydro-2H-pyran-4-yl)carbamate (**D1**) ... 19

3. Sonication Systems ... 21

4. Synthesis and Analysis of Polymers ... 22

4.1. Synthesis of Linear Polymers (LPs) ... 22

4.2. Synthesis of Network Core-Structured Star Polymers (NCSPs) ... 24

4.3. Synthesis of Mechanophore-free Network Core-Structured Star Polymers (FNCSPs) ... 29

4.4. Synthesis of Drug-Containing Network Core-Structured Star Polymers (NCSPs) ... 30

5. Supplementary Tables ... 35

6. References ... 36

1. Experimental Section

1.1. Materials

All chemical reagents and solvents were used without further purification unless otherwise stated. 2-Hydroxyethyl disulfide (technical grade, Sigma-Aldrich), methacryloyl chloride (97%, Sigma-Aldrich), triethylamine (TEA, ≥99%, Sigma-Aldrich), dichloromethane (CH_2Cl_2, anhydrous, ≥99.8%, Sigma-Aldrich), 4-(dimethylamino)pyridine (DMAP, >99.0%, TCI Deutschland GmbH), toluene (≥99.5%, Sigma-Aldrich), 1,4-dioxane (99.8%, Sigma-Aldrich), tris(2-carboxyethyl)phosphine hydrochloride (TCEP, ≥98%, Sigma-Aldrich), 4-cyano-4-(phenylcarbonothioylthio)pentanoic acid (CPA, RAFT agent, Sigma-Aldrich), ethylenebis(2-bromoisobutyrate) (97%,SigmaAldrich), bis[2(2'bromoisobutyryloxy)ethyl]-disulfide (98%, Sigma-Aldrich), $CuBr_2$ (99%, Sigma-Aldrich), Me_6TREN (97%, Sigma-Aldrich), lithium bis(trimethylsilyl)amide solution (LHMDS, 1.0 M in THF), 4-(bromomethyl)benzoic acid (97%, Sigma-Aldrich), *N,N'*-disuccinimidyl carbonate (DSC, ≥95%, Sigma-Aldrich), doxorubicin hydrochloride (Dox, Pharmaceutical Secondary Standard, Sigma-Aldrich) were used as received. 1,6-Hexanediol dimethacrylate (≥90%, Sigma-Aldrich) and Poly(ethylene glycol) methyl ether methacrylate (PEGMEMA, M_n ~ 300 Da, Sigma-Aldrich) were purified by a column of activated basic Al_2O_3 to remove the inhibitor. 2,2'-Azobis(2-methylpropionitrile) (AIBN, 98%) was obtained from Sigma-Aldrich and recrystallized twice from MeOH. Dialysis membranes (3.5 kDa MWCO) were obtained from Spectrum Labs. Centrifugal filter (3000 MWCO) was obtained from Sartorius. HeLa cell line was obtained from ATCC: The Global Bioresource Center. Ultrapure Milli-Q water (18.2 MΩ·cm) was used for all experiments.

1.2. Methods

1.2.1. General Instrumentation

^1H and ^{13}C NMR spectra were recorded at room temperature in $CDCl_3$ on a 400 MHz Bruker Avance 400 spectrometer (^{13}C: 101 MHz). The chemical shifts are reported in δ units using residual protonated solvent signals as internal standard[1] (^1H: $CDCl_3$ ($^{\delta}H$ = 7.26 ppm), ^{13}C: $CDCl_3$ ($^{\delta}C$ = 77.16 ppm)). The following abbreviations were used: s = singlet, d = doublet, t = triplet, q = quartet, sept. = septet, dd = doublet of doublets etc., m = multiplet. Coupling constants (*J*) were given in Hz and refer to the respective H, H-couplings.

TLC were performed on Merck TLC Silica gel 60 F_{254} TLC plates with a fluorescence indicator employing a 254 or 365 nm UV hand lamp for visualization. Silica gel for chromatography (40-63 µm) was used for flash column chromatography.

Gel permeation chromatography (GPC/SEC) with THF (HPLC grade) was performed using a HPLC pump (PU-2080plus, Jasco) equipped with a refractive index detector (RI-2031plus, Jasco). The sample solvent contained 250 mg·mL^{-1} 3,5-di-*t*-4-butylhydroxytoluene (BHT, ≥99%, Fluka) as internal standard. One pre-column (8×50 mm) and four SDplus gel columns (8×300 mm, SDplus, MZ Analysentechnik) were applied at a flow rate of 1.0 mL·min^{-1} at 20 °C. The diameter of the gel particles was 5 µm, the nominal pore widths were 50, 102,103, and 104 Å. Calibration was achieved using narrowly distributed poly(methyl methacrylate) (PMA) standards (Polymer Standards Service). Molar masses ($M_{n,\mathrm{GPC}}$ and $M_{w,\mathrm{GPC}}$) and molar mass distributions (M_w/M_n) were calculated by using the PSS WinGPC UniChrom software (Version 8.1.1).

ESI MS: micrOTOF-Q II™ ESI-Qq-TOF mass spectrometer system (BRUKER).

Transmission electron microscopy (TEM) micrographs were captured on a LIBRA®120 transmission electron microscope (Carl Zeiss) with an accelerating voltage of 120 kV and images were recorded using a Gatan Ultra Scan 1000. Transmission electron microscopy (TEM) sample preparation: one drop (~ 10 µL) of sample was deposited onto carbon-coated copper grid, then air-dried.

Dynamic light scattering (DLS) was measured on a Zetasizer instrument (Zetasizer Ultra, Malvern). Samples were dispersed in H_2O/DMSO (4:1, v/v) and then the mixture was transferred to a disposable plastic cell.

Ultra-high performance liquid-chromatography (UHPLC) system: ACQUITY UPLC I-Class System (Waters) with the compatible ACQUITY UPLC PDA eλ Detector and ACQUITY QDa detector (Waters). Solvents: A= water (contained 0.1% TFA), B= acetonitrile (contained 0.1% TFA); Flow= 0.4 mL·min^{-1}; Gradient (B): 0-1 min (10%), 1-5 min (10-90%), 5-7 min (90%), 7-10 min (90-10%).

1.2.2. Fluorescence Spectroscopy

Fluorescence spectra were collected by a SpectraMax iD3 multi-mode microplate reader (Molecular Devices) at room temperature. For fluorescence spectra measurements of the mixture of **B2** and polymers, samples were excited at 330 nm. The spectral bandwidths were set to 10 nm (380-750 nm) for emission. For obtaining the standard curve of **B1**, the fluorescence value was collected at emission wavelength of 550 nm. The fluorescence spectra measurements of NCSP-Dox, samples were excited at 485 nm. The spectral bandwidths were set to 5 nm (525 ~ 750 nm) for emission. For obtaining the standard curve of Furan-Dox (**D1**), the fluorescence value was collected at emission wavelength of 595 nm. The samples were kept for 72 h to complete the downstream release reactions at room temperature before the fluorescence measurements. The integration time was 0.1 s and all spectroscopic measurements were carried out with the pureGrade™ 96-wells plate purchased from BRAND GmbH. For the sonicated solution of NCSPs, filtration steps were needed before the fluorescence measurements. Filtration was carried out as follows: Centrifugation for 3 times at RCF 8000 ×g for 10 min, then the suspensions were filtered through a centrifugal filter (3,000 MWCO) by centrifugation at 5,000 rpm.

1.2.3. Sonication Experiments

20 kHz sonicator and 1.5 MHz HIFU setup performed ultrasound sonication experiments (**Figure S1**).

20 kHz: Sonication experiments were carried out using a Qsonica Q125 ultrasonic system with a 3.2 mm probe, f = 20 kHz. Pulsed sonication (2 s on, 1 s off) was used. 10 mg polymers was dissolved in 1 mL H_2O/DMSO (4:1, v/v), then injected into a cooled Eppendorf tube (immersed in an ice-water bath). Then, the mixture was exposed to sonication for 2 min, 5 min, 10 min, 30 min, 60 min and 90 min.

1.5 MHz HIFU: Ultrasound sonication experiments were performed with a home-built HIFU setup. The core devices include waveform generator (33511B, Keysight Technologies), RF amplifier (AG1021, T&C Power Conversion, Inc.) and transducers (1.5 MHz, Precision Acoustics Ltd., UK). A 0.5 mm needle hydrophone (Precision Acoustics Ltd., UK) was used for locating the transducer focal point. Custom-made motorized 3D-manipulator/positioning system for controlling the well plate submerged in water was employed. Pulsed sonication (2 s on, 1 s off) was used. 10 mg polymers was dissolved in 1 mL H_2O/DMSO (4:1, v/v), then the solution was added into the 24 well plate that with an acoustically transparent base made of ultra-thin film (lumox® multiwall 24, SARSTEDT). Samples exposed to constant sonication for 3 min, 10 min, 15 min and 20 min.

1.2.4. Cell Imaging

HeLa cells used for imaging were cultured in Dulbecco's Modified Eagle's Medium (DMEM) containing 10% heat-inactivated fetal bovine serum (FBS) supplemented with 100 U/mL of penicillin and 100 µg/mL streptomycin at 37 °C under a humidified atmosphere containing 5% CO_2. The HeLa cells were seeded in an ibidi µ-Slide 8 Wells (with glass bottom) at a density of 2.5×10^5 cells per well in 500 µL culture medium. After 24 h, HeLa cells were incubated with different concentrated sonicated samples or non-sonicated samples in PBS for 2 h at 37 °C, then washed with phosphate-buffered saline (PBS) three times. Then, PBS was added into the wells (500 µL per well), 2 µL (1 mg/mL) Calcein AM and 2 µL (1 mg/mL) propidium iodide were transferred to the wells mixed for 5min. PBS was used to wash out the free cell imaging agents. Then the fluorescence imaging of cells was performed on a confocal laser scanning microscope (STP8, Leica) (confocal excitation: Calcein AM: 496 nm, propidium iodide: 561 nm) and analyzed by ImageJ.

1.2.5. MTS Proliferation Assays

HeLa cells were used to evaluate the cytotoxicity of different samples. HeLa cells were cultured in a basal medium containing DMEM (supplemented with 10% fetal bovine serum and 1% antibiotics/antimycotics) at 37 °C. Actual cell viability was monitored by using a tetrazolium compound 3-(4,5-dimethylthiazol-2-yl)-5-(3-carboxymethoxyphenyl)-2-(4-sulfophenyl)-2H-tetrazolium (inner salt, MTS reagent) and a chemical electron acceptor dye (phenazine ethosulfate; PES) (Promega, Germany) using an assay according to the manufacturer's instructions. Briefly, approximately 5,000 cells in 100 µL of medium were seeded into 96-well plates. After overnight incubation, the culture medium in 96-well plates was removed and exchanged with fresh medium (100 µL) containing different concentrated testing samples. Control cultures were treated with DMSO alone. The final concentration of DMSO in the medium did not exceed 0.5%. After 48 h incubation, the cell culture media were removed, the cells were washed with 100 µL PBS buffer, and then 20 µL MTS reagent with 100 µL fresh medium was added to the cells. The mixture of MTS reagent with cell culture medium served as negative control. The

resulting solution was mixed thoroughly, and the absorbance was monitored using a microplate spectrophotometer at 490 nm (Synergy™ HT microplate reader, BioTek Instruments). MTS signals were used for survival and proliferation determination. All the sample cultures were performed at least in triplicates.

1.2.6. Quantification Method of Disulfide Activation Percentage

The disulfide activation percentage was calculated by a simple quantification method, as the following description:

For linear polymers: 10 mg linear polymers was dissolved in 1 mL H_2O/DMSO (4:1, v/v), then pipette 100 μl polymers solution into a tube, TCEP (0.3 mg) was added to the mixture to cleave all disulfide bonds within the polymers. Subsequently, DI water was used to wash the LPs two times (the mixture were filtered through a centrifugal filter (3,000 MWCO) by centrifugation at 5,000 rpm). Next, **B2** (0.1 mg) dissolved in the H_2O/DMSO (1 mL) mixed with the filtered polymers residual. After 3 d reaction that make sure all the thiols reacted with B2, the fluorescence intensity at 550 nm of the solution was measured. We take the obtained fluorescence intensity at 550 nm as the maximum value (F_m). Then, **B2** (0.1 mg) dissolved in the H_2O/DMSO (1 mL) mixed with the sonicated polymers residual by the same method. We collected the fluorescence intensity at 550 nm (F_s) from the sonicated samples. When measuring the fluorescence intensity, the samples were diluted by the same factor to make sure the F_s and F_m were in the same range according to the standard curve of fluorescence molecule B1 (**Figure S13**). Finally, we take the value of F_s / F_m as the results of disulfide activation percentage.

For star polymers: 10 mg star polymers was dissolved in 1 mL H_2O/DMSO (4:1, v/v), then then pipette 100 μL polymers solution into a tube, TCEP (1.3 mg) was added to the mixture to cleave all disulfide bonds within the polymers. Subsequently, DI water was used to wash the star polymers two times (the mixture were filtered through a centrifugal filter (3,000 MWCO) by centrifugation at 5,000 rpm). Next, **B2** (0.5 mg) dissolved in the H_2O/DMSO (1 mL) mixed with the polymers residual. After 3 d reaction that make sure all the thiols reacted with B2, the fluorescence intensity at 550 nm of the solution was measured. We take this obtained fluorescence intensity at 550 nm as the maximum value (F_m). Then, **B2** (0.5 mg) dissolved in the H_2O/DMSO (1 mL) mixed with the sonicated polymers residual by the same method. We collected the fluorescence intensity at 550 nm (F_s) from the sonicated samples. When measuring the fluorescence intensity, the samples were diluted by the same factor to make sure the F_s and F_m were in the same range according to the standard curve of fluorescence molecule B1 (**Figure S13**). Finally, we take the value of F_s / F_m as the results of disulfide activation percentage.

In addition, for the quantification of the concentration of activated thiols or cleaved disulfide bonds, our established method can be used.[2]

2. Synthetic Procedures and Characterization Data

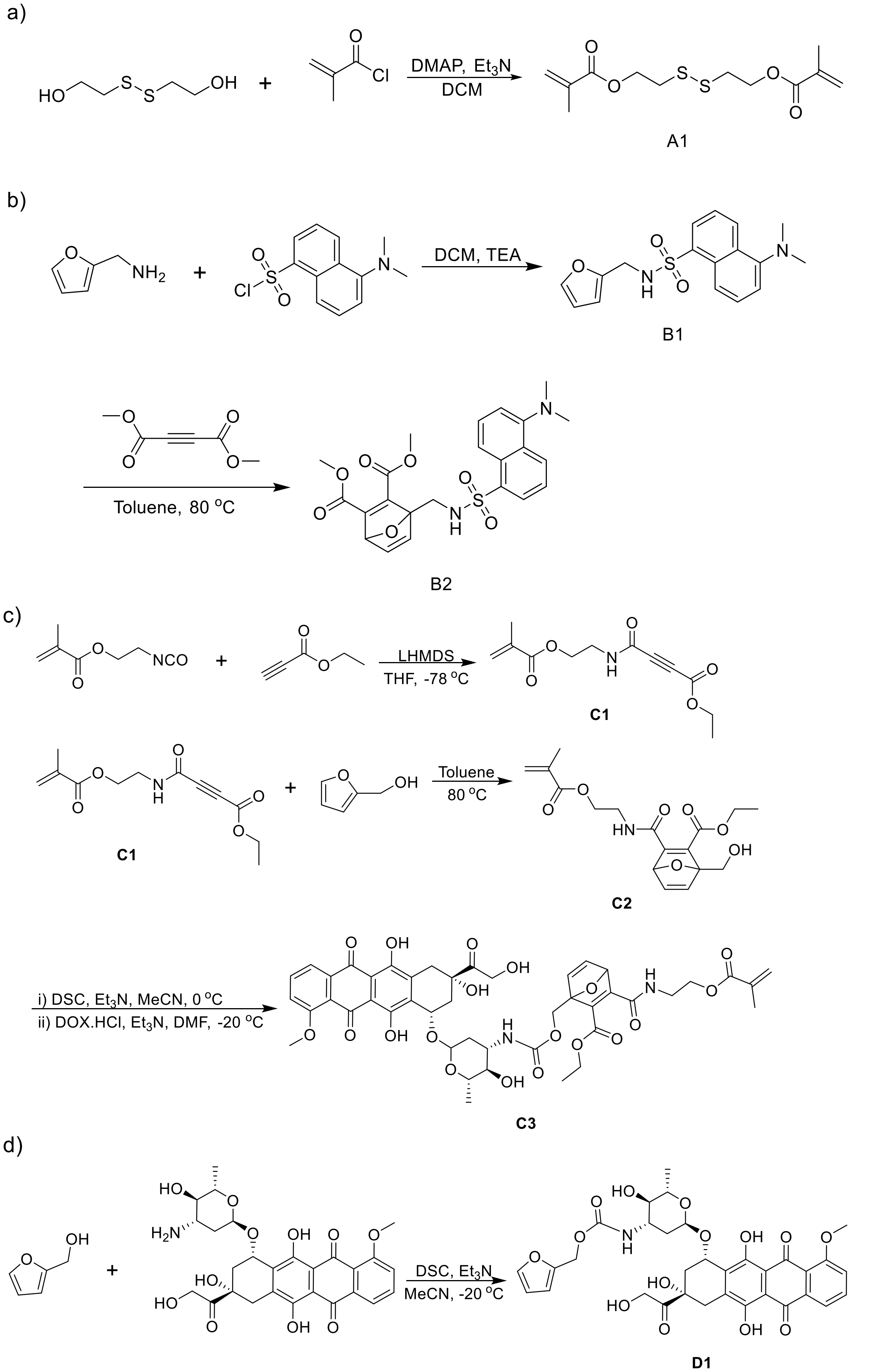

Scheme S1. Synthesis of **A1**, **B2**, **C3**, and **D1**.

2.1. Disulfanediylbis(ethane-2,1-diyl) bis(2-methylacrylate) (**A1**)

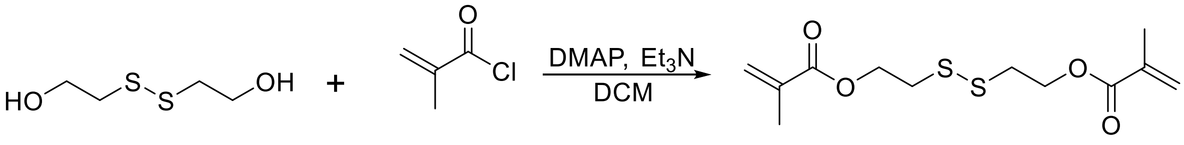

A1

2-Hydroxyethyl disulfide (2.0 g, 13 mmol, 1 equiv.) and TEA (3.64 mL, 26 mmol, 2.0 equiv.) were added in THF (30 mL) with a constant flow of N_2. The mixture was subject into the ice-water bath to keep the temperature at 0 °C. Afterwards, a solution of methacryloyl chloride (2.54 mL, 26 mmol, 2.04 equiv.) and 15 mL CH_2Cl_2 was added dropwise (15 mL/h) to the reaction mixture via an injection pump. The reaction mixture was stirred until complete consumption of the starting material as indicated by TLC. Subsequently, the reaction mixture was washed three times with brine and three times with H_2O. Afterwards, the crude product was purified by column chromatography on silica gel (hexane:EtOAc = 5:1) to give compound **A1** (3.2 g, 85% yield) as a colourless oil. **^{1}H NMR (400 MHz, $CDCl_3$):** δ (ppm): 6.12 (sept, J=1.6 Hz, 2H), 5.58 (sept, J=1.6 Hz, 2H), 4.4 (t, J=6.8 Hz, 4H), 2.97 (t, J=6.8 Hz, 4H), 1.94 (s, J=1.2 Hz, 6H). **^{13}C NMR (101 MHz, $CDCl_3$):** δ (ppm): 167.23, 136.12, 126.13, 62.6, 37.39, 18.4. **ESI-MS (*m/z*)** for $C_{12}H_{18}O_4S_2$ expected $[M+Na]^+$: 313.0539, Found for $[M+Na]^+$: 313.0590.

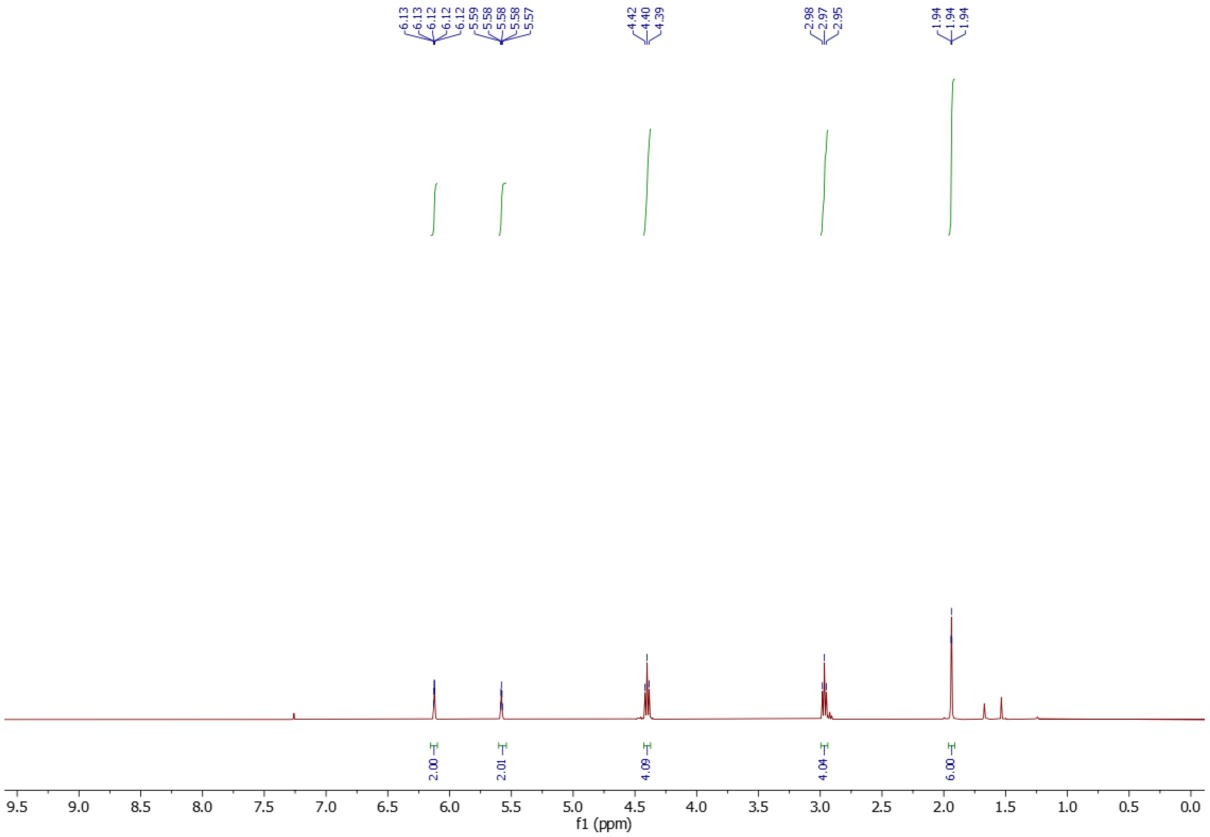

^{1}H NMR spectrum of disulfanediylbis(ethane-2,1-diyl) bis(2-methylacrylate) (**A1**).

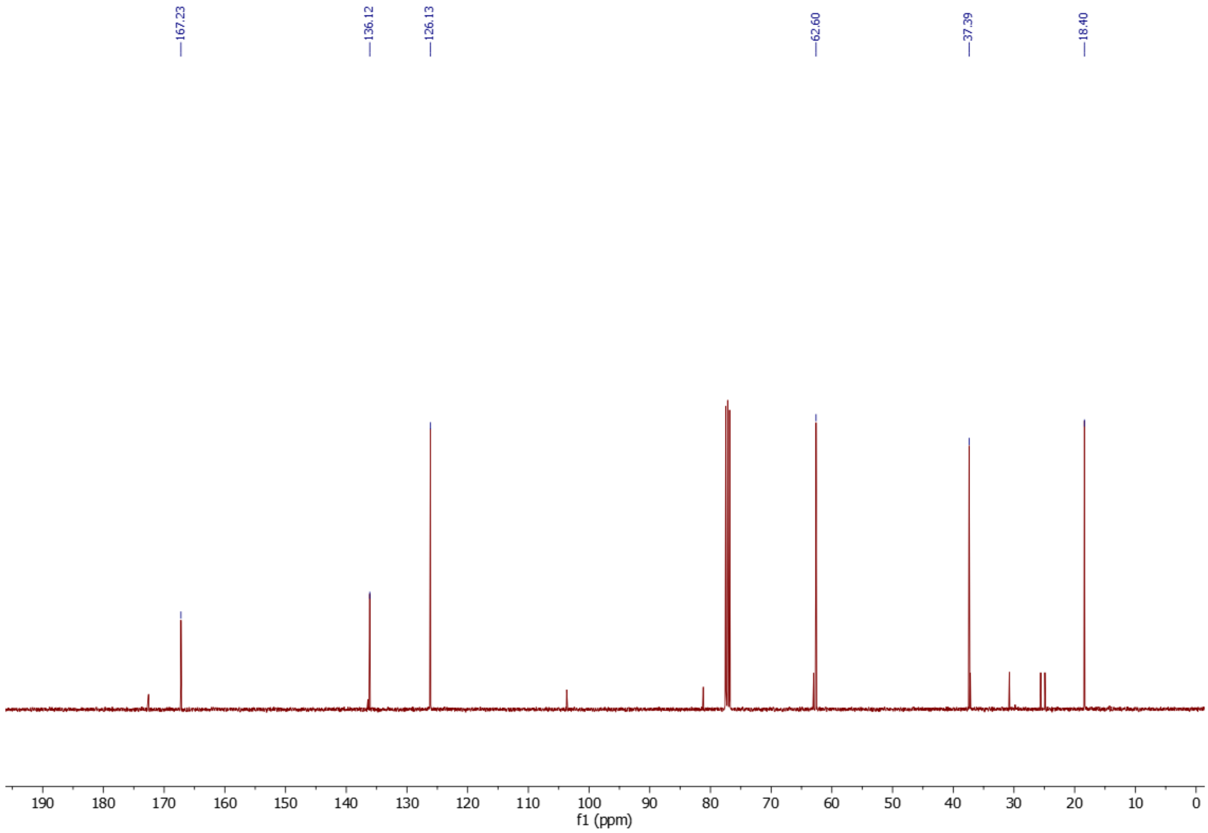

^{13}C NMR spectrum of disulfanediylbis(ethane-2,1-diyl) bis(2-methylacrylate) (**A1**).

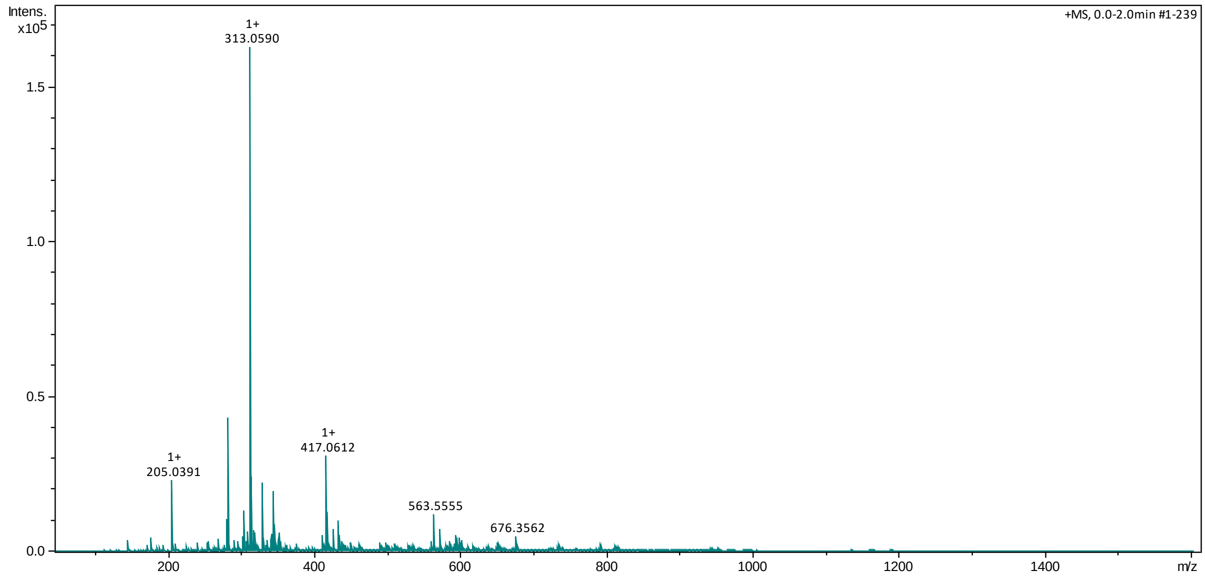

ESI-MS (*m/z*) of disulfanediylbis(ethane-2,1-diyl) bis(2-methylacrylate) (**A1**).

2.2. 5-(dimethylamino)-N-(furan-2-ylmethyl)naphthalene-1-sulfonamide (**B1**)

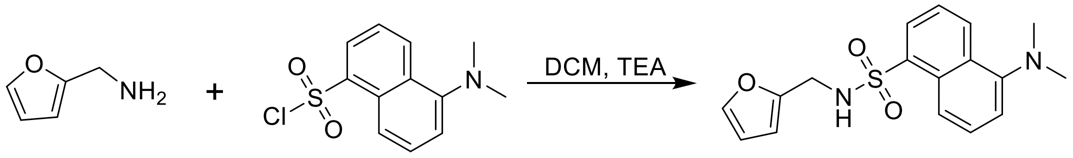

B1

5-(Dimethylamino)-*N*-(furan-2-ylmethyl)naphthalene-1-sulfonamide is synthesized according to a modified literature reaction (Scheme S1).[3] A mixture of 5-(dimethylamino)naphthalene-1-sulfonyl chloride (1.00 g, 3.70 mmol, 1.0 equiv.) and Et_3N (1.0 mL, 7.2 mmol, 2.0 equiv.) in CH_2Cl_2 (20 mL) is added into a solution of furfurylamine (380 mg, 3.9 mmol, 1.05 equiv.) in CH_2Cl_2 (5 mL) via syringe under N_2 protection. The resulting solution is stirred for 5 h at room temperature and poured into 1.0 mM pH 7 phosphate buffer (30 mL). Afterwards, the crude product was purified by column chromatography on silica gel (hexane:EtOAc = 5:1) to give compound **B1** (1.16 g, 95% yield) as a yellow-green oil. **^{1}H NMR (400 MHz, $CDCl_3$):** δ (ppm): 8.50 (d, 1H, J = 8.0 Hz), 8.25-8.20 (m, 2H), 7.54-7.45 (m, 2H), 7.16 (d, 1H, J = 8.0 Hz), 7.01 (dd, 1H, J = 2.0 Hz, 0.8 Hz), 6.02 (dd, 1H, J = 3.2 Hz, 2.0 Hz), 5.87 (dd, 1H, J = 3.2 Hz, 0.6Hz), 5.19 (m, 1H), 4.12 (d, 2H, J = 6.0 Hz), 2.87 (s, 6H). **^{13}C NMR (101 MHz, $CDCl_3$):** δ (ppm): 151.95, 149.41, 142.20, 134.72, 130.50, 129.84, 129.60, 128.42, 123.13, 118.61, 115.14, 110.14, 107.98, 45.42, 40.20. **ESI-MS (*m/z*)** for $C_{17}H_{18}N_2O_3S$ expected $[M+H]^+$: 331.1111, Found for $[M+H]^+$: 331.1061.

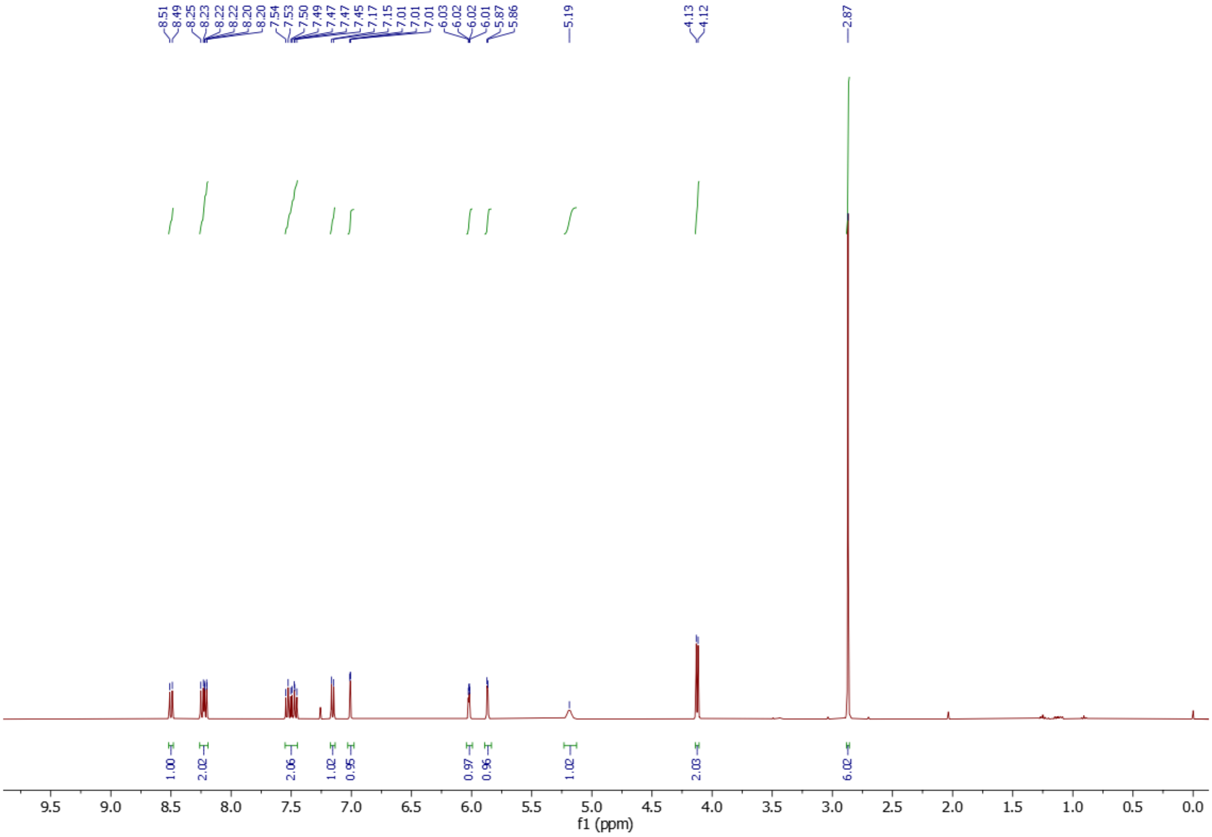

^{1}H NMR spectrum of 5-(dimethylamino)-N-(furan-2-ylmethyl)naphthalene-1-sulfonamide (**B1**).

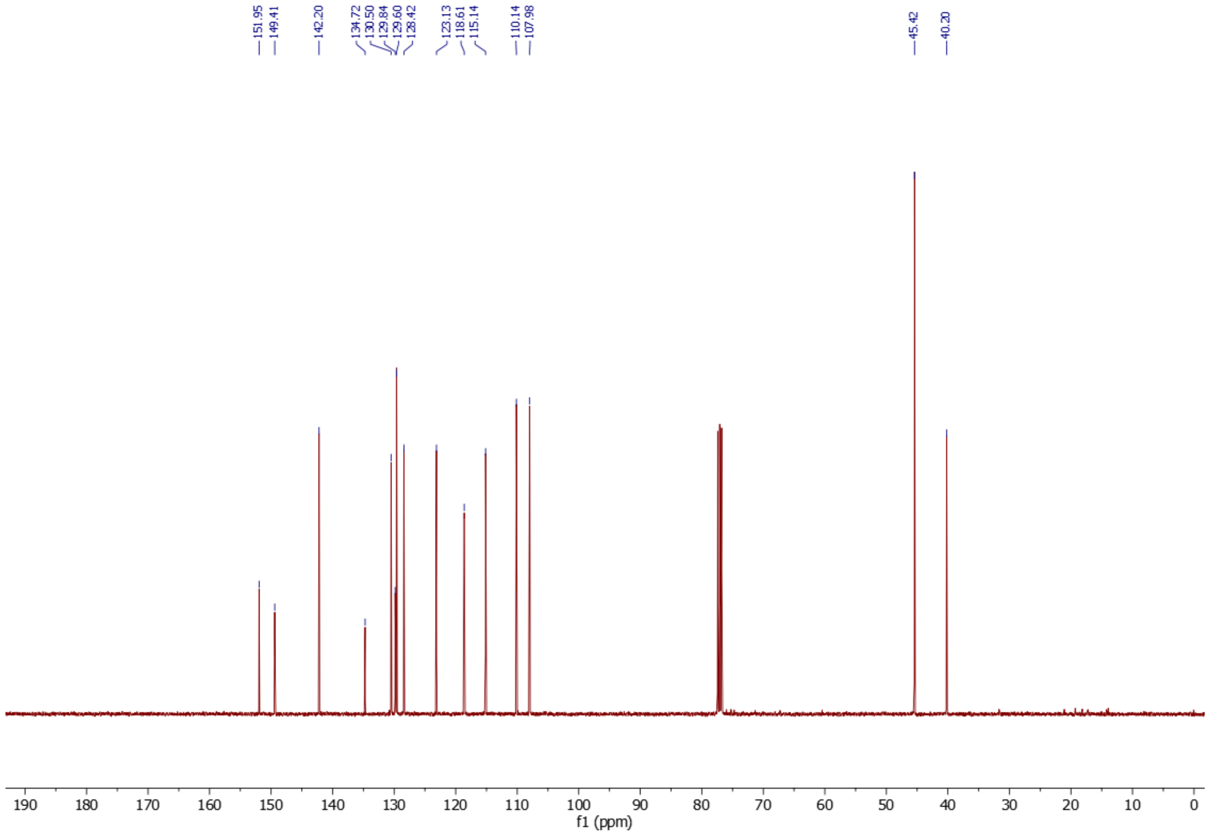

^{13}C NMR spectrum of 5-(dimethylamino)-N-(furan-2-ylmethyl)naphthalene-1-sulfonamide (**B1**).

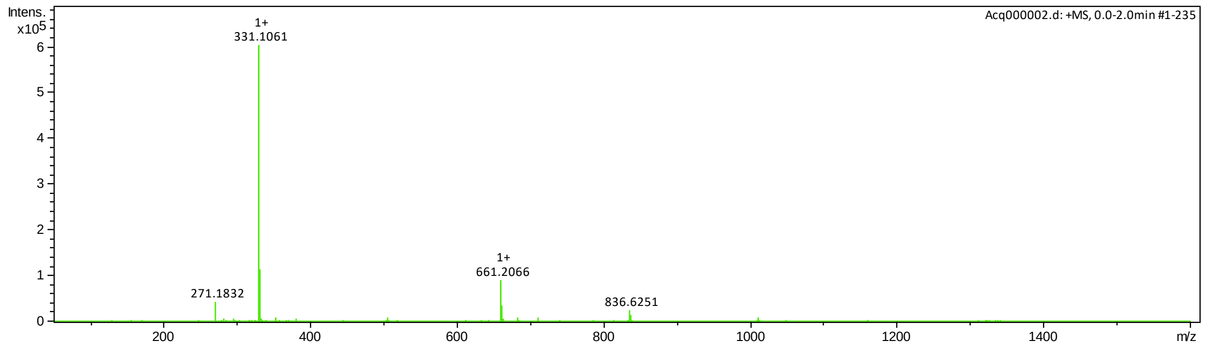

ESI-MS (*m/z*) of 5-(dimethylamino)-N-(furan-2-ylmethyl)naphthalene-1-sulfonamide (**B1**).

2.3. Dimethyl 1-(((5-(dimethylamino)naphthalene)-1-sulfonamido)methyl)-7-oxabicyclo[2.2.1]hepta-2,5-diene-2,3-dicarboxylate (**B2**)

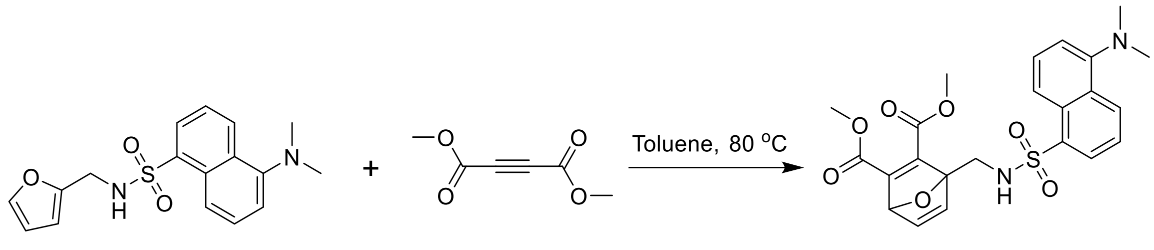

B1 **B2**

Dimethyl acetylenedicarboxylate (0.71 g, 5.0 mmol, 2.5 equiv.) and 5-(dimethylamino)-N-(furan-2-ylmethyl)naphthalene-1-sulfonamide (0.66 g, 2.0 mmol, 1.0 equiv.) are dissolved in 8 mL dry toluene. The solution was stirred at 70 °C for 48 h. After the solvent is removed *in vacuo*, the crude product was purified by column chromatography on silica gel (hexane:EtOAc = 2:1) to give compound **B2** (0.83 g, 88% yield) as a yellow oil. **^{1}H NMR (400 MHz, CDCl$_3$):** δ (ppm): 8.55 (d, 1H, J = 8.4 Hz), 8.28-8.23 (m, 2H), 7.59-7.51 (m, 2H), 7.19 (d, 1H, J = 7.6 Hz), 7.13 (dd, 1H, J = 5.2 Hz, 2.0 Hz), 6.89 (d, 1H, J = 5.2 Hz), 5.57 (d, 1H, J = 2.0 Hz), 5.25 (t, 1H, J = 6.0 Hz), 3.78 (s, 3H), 3.75-3.69 (m, 4H), 3.60 (dd, 1H, J = 14.0 Hz, 5.6 Hz), 2.89 (s, 6H). **^{13}C NMR (101 MHz, CDCl$_3$):** δ (ppm): 163.72, 162.72, 153.53, 152.02, 151.96, 144.88, 142.71, 134.32, 130.68, 129.92, 129.72, 129.60, 128.59, 123.19, 118.65, 115.31, 95.80, 83.96, 52.51, 52.42, 45.43, 41.93, 40.99. **ESI-MS (*m/z*)** for $C_{23}H_{24}N_2O_7S$ expected [M+H]$^+$: 473.1377, Found for [M+H]$^+$: 473.1349.

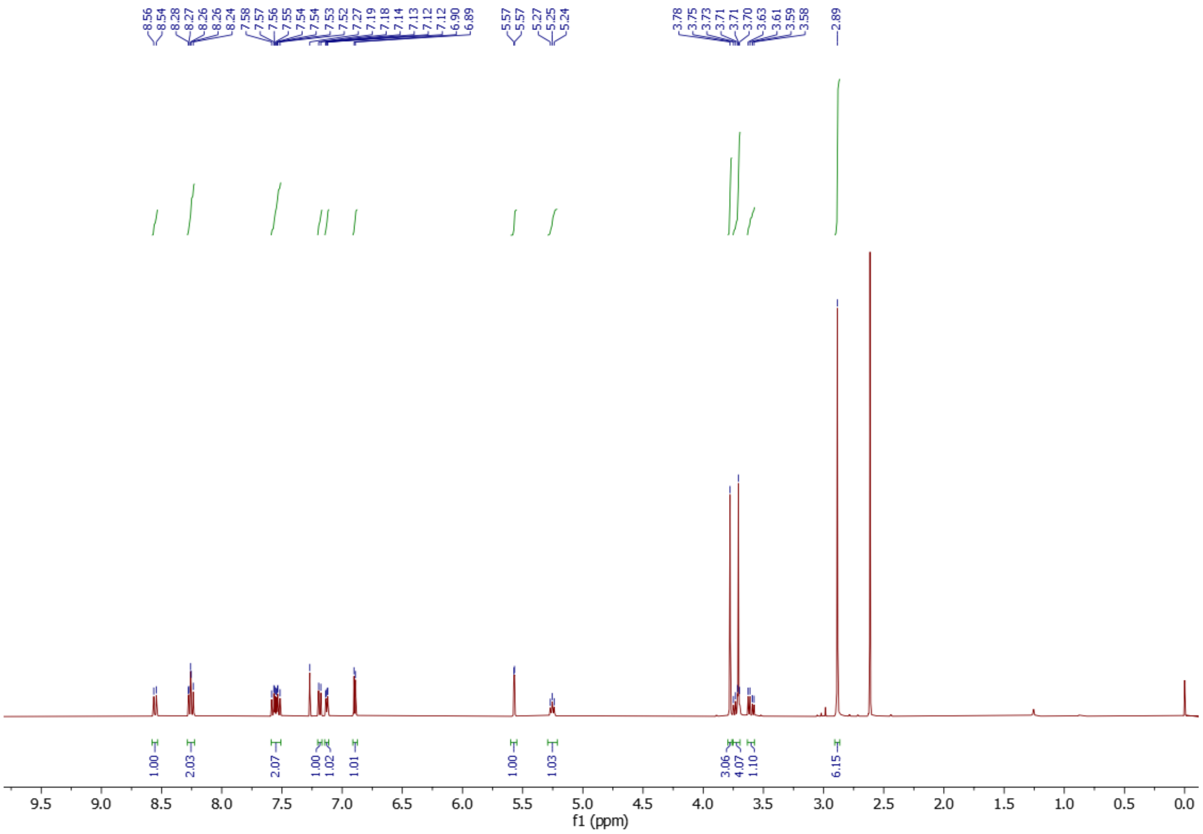

^{1}H NMR spectrum of dimethyl 1-(((5-(dimethylamino)naphthalene)-1-sulfonamido)methyl)-7-oxabicyclo[2.2.1]hepta-2,5-diene-2,3-dicarboxylate (**B2**).

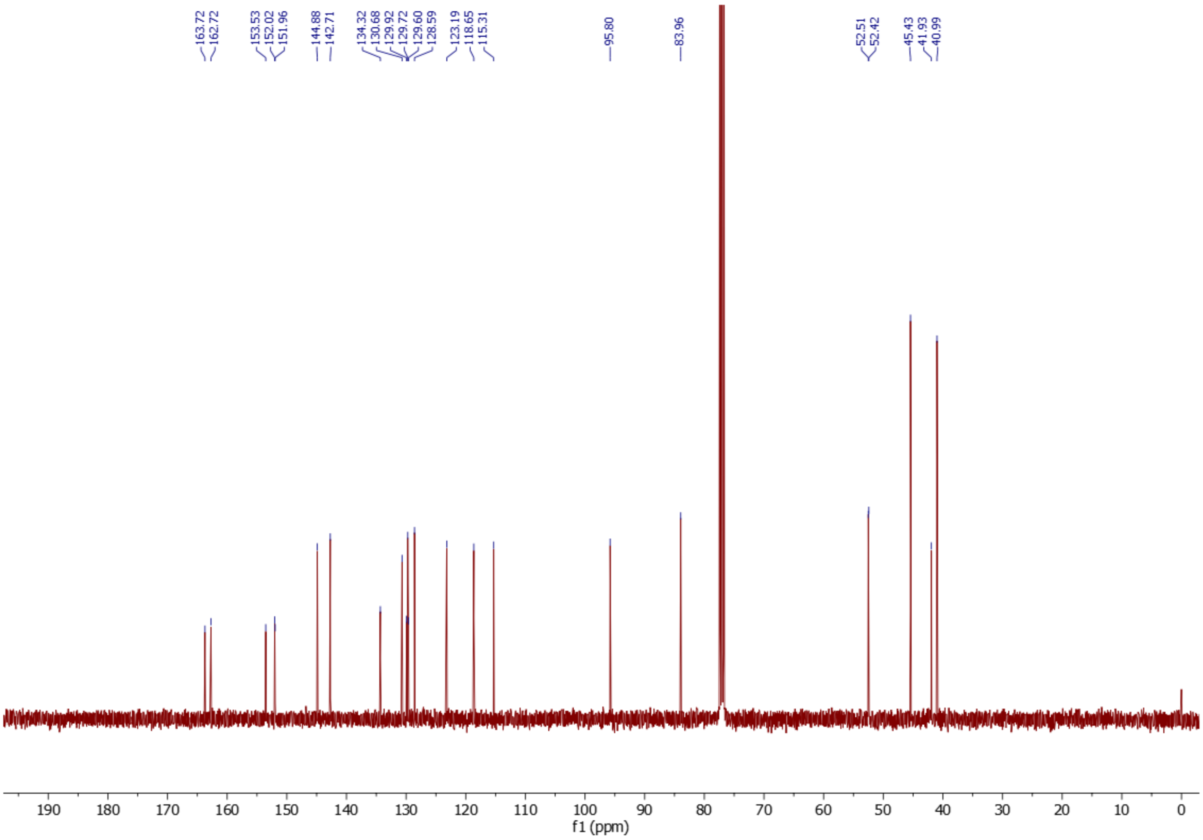

^{13}C NMR spectrum of dimethyl 1-(((5-(dimethylamino)naphthalene)-1-sulfonamido)methyl)-7-oxabicyclo[2.2.1]hepta-2,5-diene-2,3-dicarboxylate (**B2**).

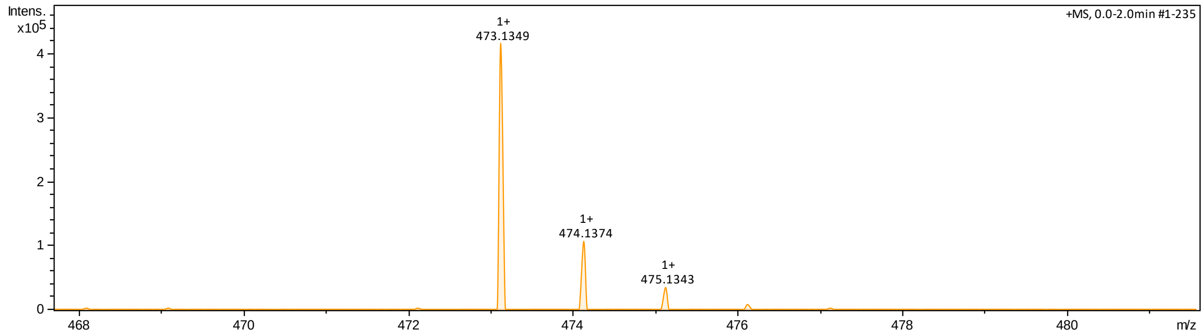

ESI-MS (*m/z*) of dimethyl 1-(((5-(dimethylamino)naphthalene)-1-sulfonamido)methyl)-7-oxabicyclo[2.2.1]hepta-2,5-diene-2,3-dicarboxylate (**B2**).

2.4. Ethyl 4-((2-(methacryloyloxy)ethyl)amino)-4-oxobut-2-ynoate (**C1**)

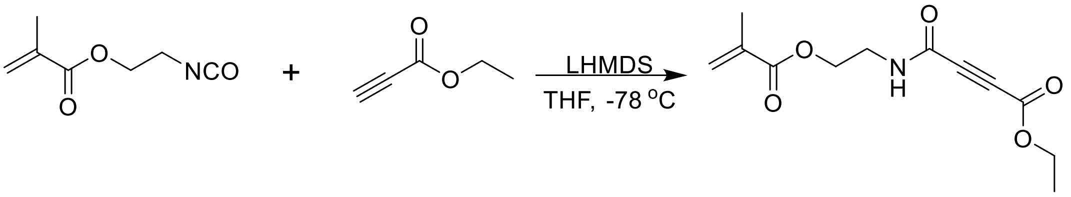

Ethyl 4-((2-(methacryloyloxy)ethyl)amino)-4-oxobut-2-ynoate **C1** was synthesized according to a publication.[4] Ethylpropiolate (4.0 mL, 39.5 mmol, 1.0 equiv.) was dissolved in THF (60 mL) and the solution was cooled down to -78 °C with a dry ice/acetone bath. Then, LHMDS (1 M in THF, 40 mL, 40 mmol, 1.01 equiv.) was added with a syringe pump over a period of 30 min. The solution was stirred for an additional 30 min at -78 °C. Then, 2-isocyanatoethyl methacrylate (5.6 mL, 39.5 mmol, 1.0 equiv.) was added dropwise with a syringe pump. After the solution was stirred for 30 min at -78 °C, it was quenched by adding a sat. aq. NH_4Cl solution (80 mL) at -78 °C. When the solution reached room temperature, H_2O (50 mL) was added and the two phases were separated. The aqueous layer was extracted with EtOAc (3×100 mL). The collected organic layers were washed with a sat. aq. $NaHCO_3$ and brine. Then, the organic layer was dried over $MgSO_4$, concentrated, and purified *via* a column chromatography (hexane:EtOAc = 10:1) to give compound **C1** (8.09 g, 81% yield) as a colourless oil. **^{1}H NMR (400 MHz, $CDCl_3$):** δ (ppm): 6.85 (t, J = 6.0 Hz, 1H), 6.10 (s, 1H), 5.61 – 5.56 (m, 1H), 4.28 – 4.20 (m, 4H), 3.61 (q, J = 5.6 Hz, 2H), 1.91 (s, 3H), 1.29 (t, J = 7.2 Hz, 3H). **^{13}C NMR (101 MHz, $CDCl_3$):** δ (ppm): 167.45, 152.34, 151.08, 135.77, 126.55, 76.93, 74.26, 63.03, 62.83, 39.28, 18.30, 13.94. **ESI-MS (*m/z*)** for $C_{12}H_{15}NO_5$ expected $[M+H]^+$: 254.1023, Found for $[M+H]^+$: 254.1030.

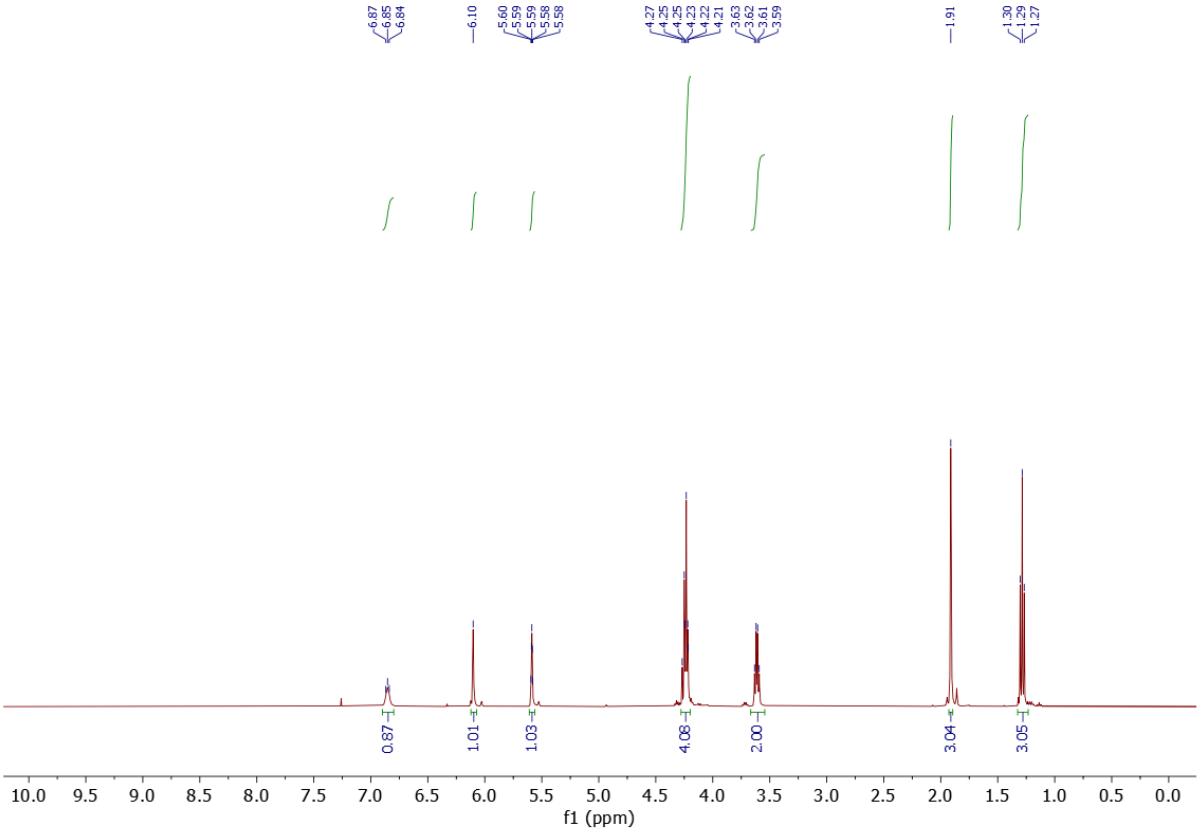

^{1}H NMR spectrum of Ethyl 4-((2-(methacryloyloxy)ethyl)amino)-4-oxobut-2-ynoate (**C1**).

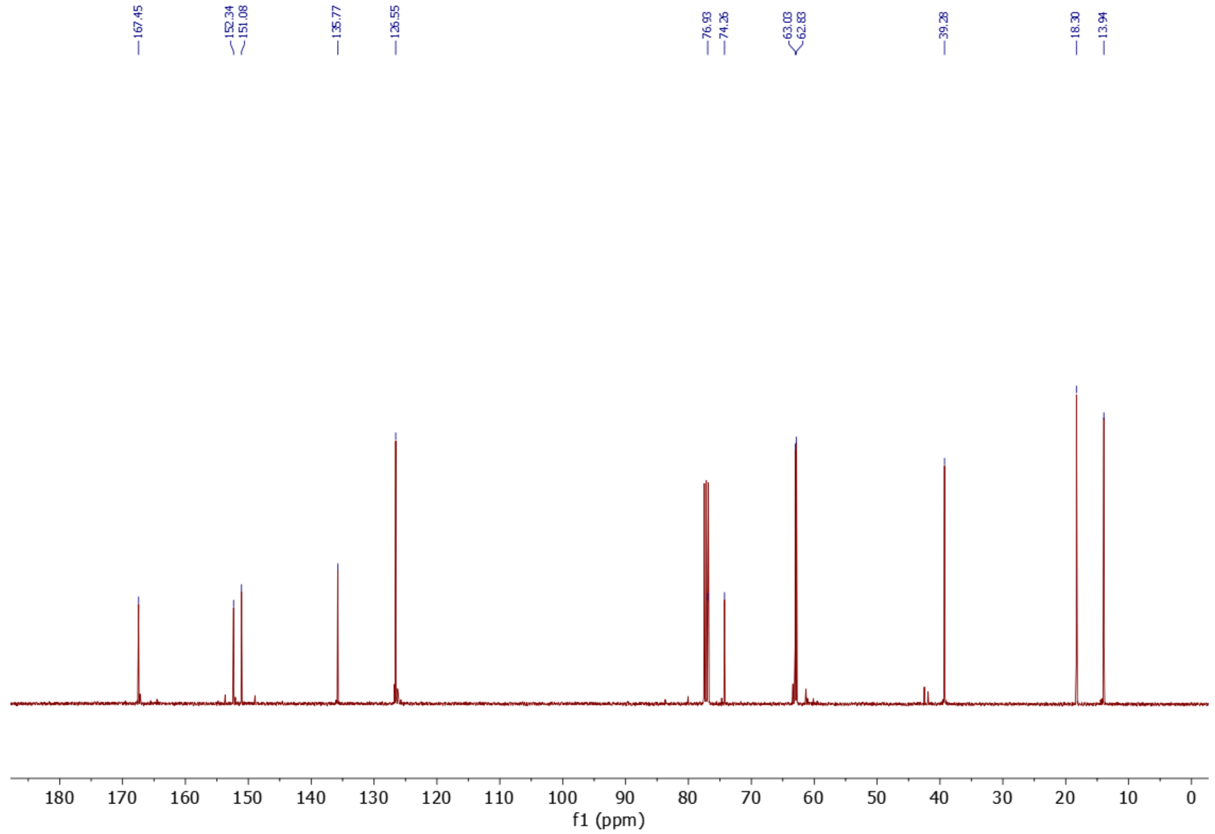

^{13}C NMR spectrum of Ethyl 4-((2-(methacryloyloxy)ethyl)amino)-4-oxobut-2-ynoate (**C1**).

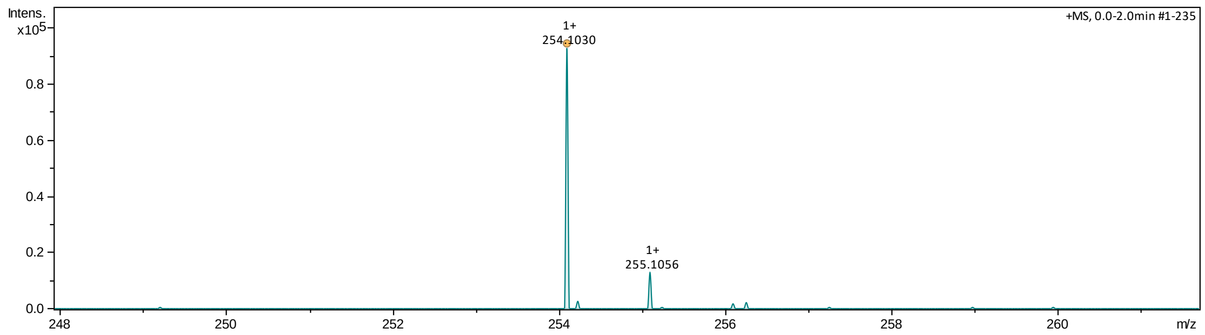

ESI-MS (*m/z*) of Ethyl 4-((2-(methacryloyloxy)ethyl)amino)-4-oxobut-2-ynoate (**C1**).

2.5. Ethyl 1-(hydroxymethyl)-3-((2-(methacryloyloxy)ethyl)carbamoyl)-7-oxabicyclo[2.2.1]hepta-2,5-diene-2-carboxylate (**C2**)

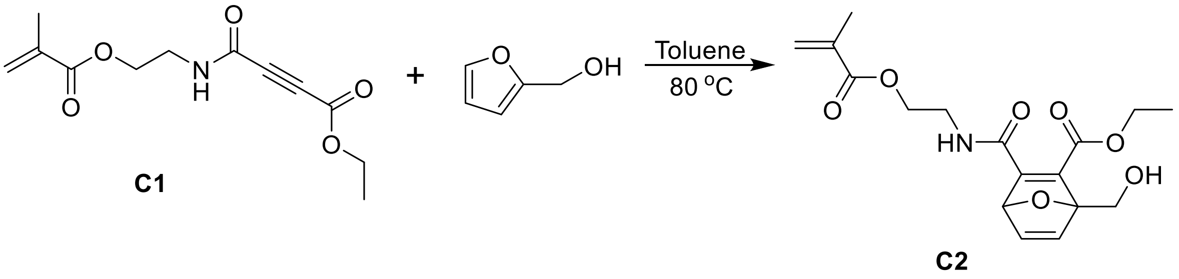

C1 (0.57 g, 2.26 mmol, 1.0 equiv.) and furan-2-ylmethanol (0.46 g, 4.7 mmol, 2.08 equiv.) are dissolved in 2.5 mL dry toluene. The solution was stirred at 80 °C for 48 h. After the solvent is removed *in vacuo*, the crude product was purified by column chromatography on silica gel (hexane:EtOAc = 2:1) to give compound **C2** (0.397 g, 50% yield) as a colorless oil. **^{1}H NMR (400 MHz, CDCl$_3$):** δ (ppm): 9.37 (t, J = 5.6 Hz, 1H), 7.23 (d, J = 5.2 Hz, 1H), 7.15 (dd, J = 5.2, 1.6 Hz, 1H), 6.17 (s, 1H), 5.64 (d, J = 1.6 Hz, 1H), 5.60 – 5.55 (m, 1H), 4.33 – 4.11 (m, 6H), 3.64 (q, J = 5.6 Hz, 2H), 1.94 (s, 3H), 1.33 (t, J = 7.2 Hz, 3H). **^{13}C NMR (101 MHz, CDCl$_3$):** δ (ppm): 167.25, 164.50, 163.15, 161.16, 150.32, 143.70, 143.39, 136.09, 126.12, 98.62, 83.75, 63.05, 62.36, 60.54, 38.82, 18.33, 14.14. **ESI-MS (*m/z*)** for $C_{17}H_{21}NO_7$ expected $[M+H]^+$: 352.1391, Found for $[M+H]^+$: 352.1363.

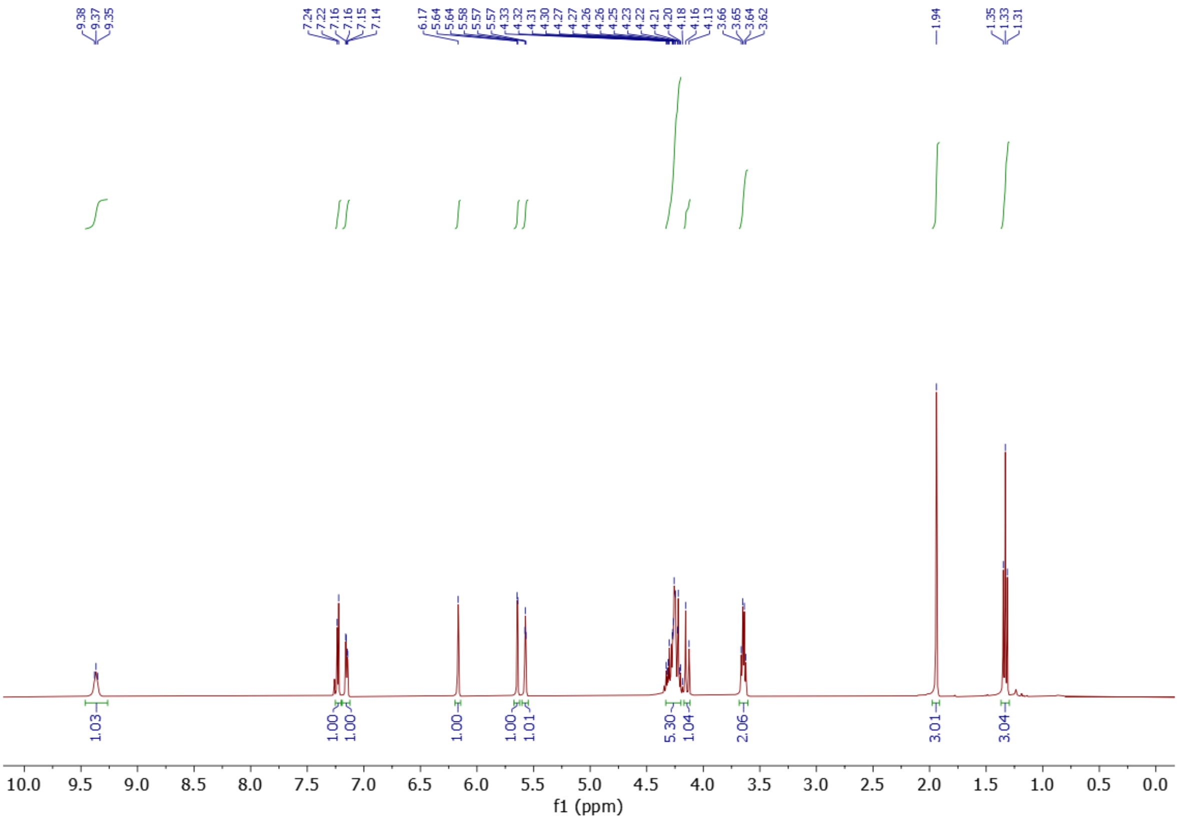

^{1}H NMR spectrum of Ethyl 1-(hydroxymethyl)-3-((2-(methacryloyloxy)ethyl)carbamoyl)-7-oxabicyclo[2.2.1]-hepta-2,5-diene-2-carboxylate (**C2**).

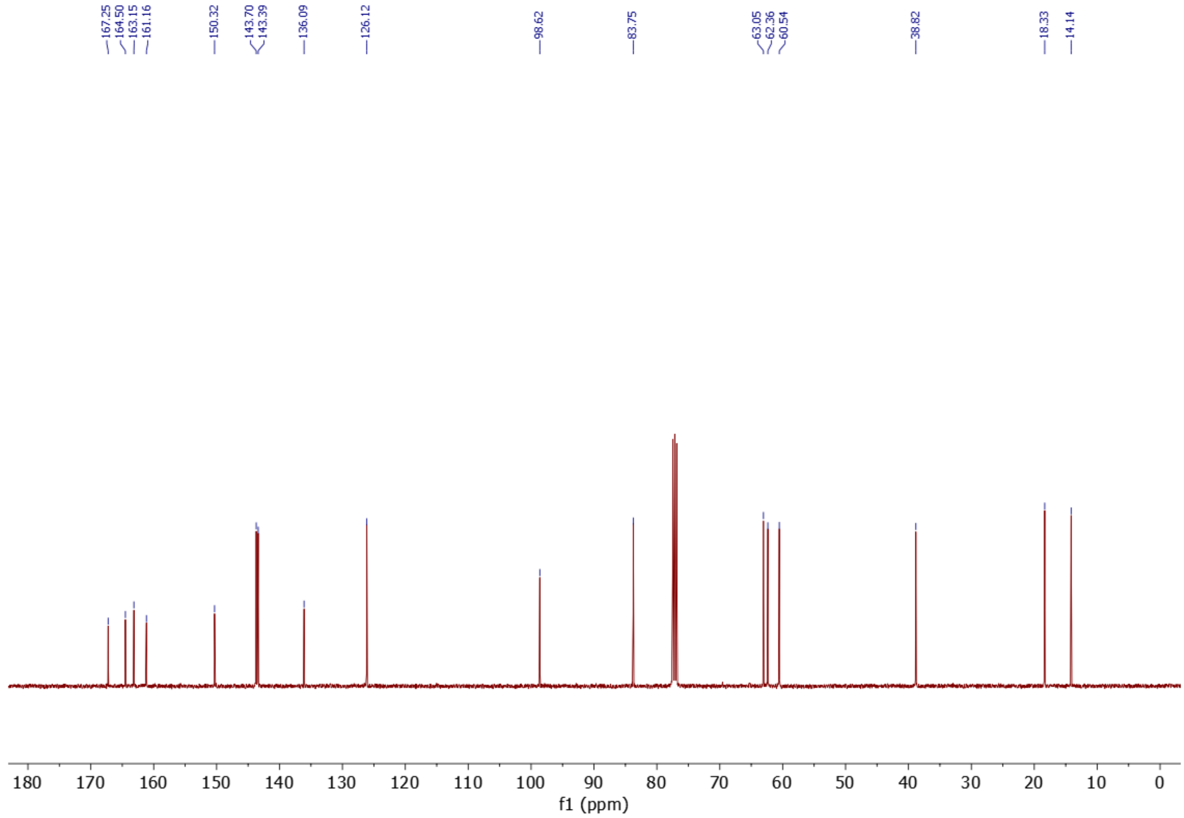

^{13}C NMR spectrum of Ethyl 1-(hydroxymethyl)-3-((2-(methacryloyloxy)ethyl)carbamoyl)-7-oxabicyclo[2.2.1]-hepta-2,5-diene-2-carboxylate (**C2**).

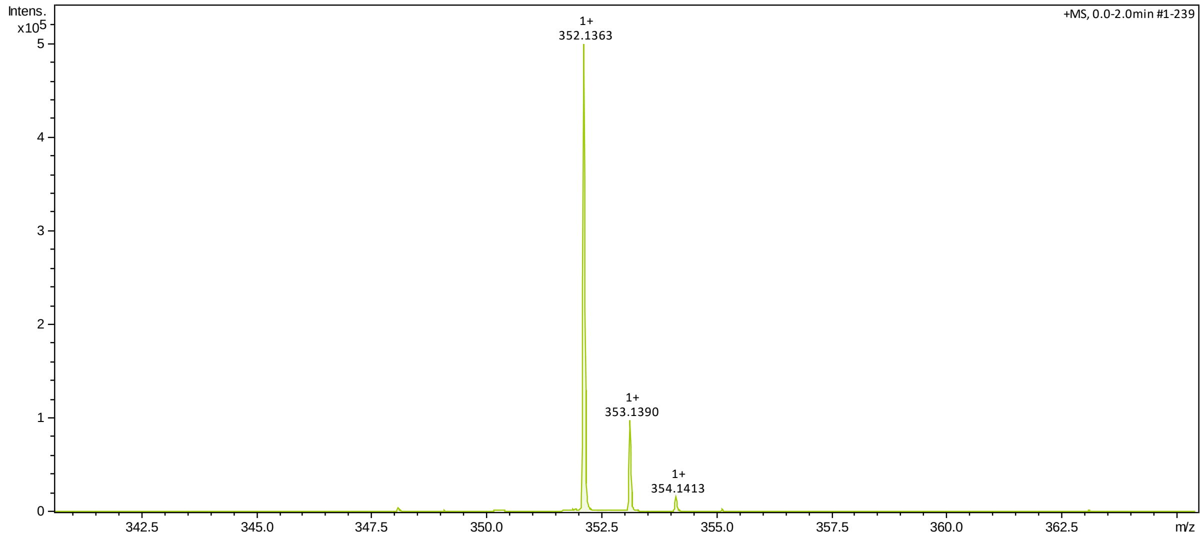

ESI-MS (*m/z*) of Ethyl 1-(hydroxymethyl)-3-((2-(methacryloyloxy)ethyl)carbamoyl)-7-oxabicyclo[2.2.1]-hepta-2,5-diene-2-carboxylate (**C2**).

2.6. Ethyl 1-(((((2S,3R,4S)-3-hydroxy-2-methyl-6-(((1S,3S)-3,5,12-trihydroxy-3-(2-hydroxyacetyl)-10-methoxy-6,11 -dioxo-1,2,3,4,6,11-hexahydrotetracen-1-yl)oxy)tetrahydro-2H-pyran-4-yl)carbamoyl)oxy)methyl)-3-((2 (methacryloyloxy)ethyl)carbamoyl)-7-oxabicyclo[2.2.1]hepta-2,5-diene-2-carboxylate (**C3**)

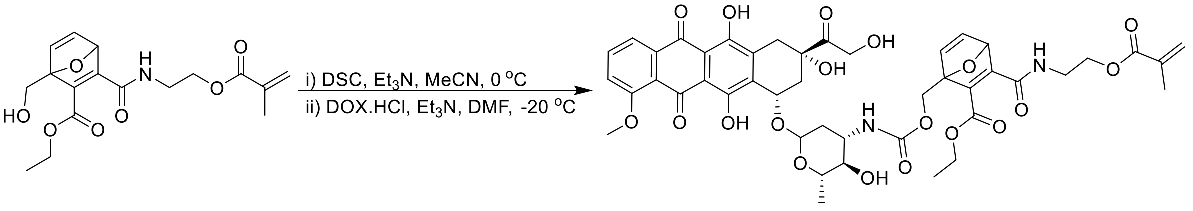

C2 **C3**

The preparation followed an adapted literature protocol.[5] *N,N'*-disuccinimidyl carbonate (132 mg, 517 µmol, 1.0 equiv.), Et_3N (90 µL, 690 µmol, 1.3 equiv.) were dissolved in MeCN (2 mL) in a round bottom flask. The mixture was cooled to 0 °C and a solution of **C2** (121 mg, 345 µmol, 0.67 equiv.) in MeCN (1 mL) was added. The reaction mixture was allowed to stir in an ice bath for 1 h. Subsequently, the mixture was dried *in vacuo* and used without further purification. The obtained material was dissolved in CH_2Cl_2 and added dropwise into a cooled (-20 °C) solution of doxorubicin hydrochloride (70 mg, 120 µmol, 0.23 equiv.) and Et_3N (92.5 µL, 709 µmol, 1.0 equiv.) in DMF (2 mL). The reaction mixture was stirred for 1 h at -20 °C and then warmed up slowly to room temperature over 3 h. Afterwards, the solvent was removed *in vacuo*, the residue redissolved in CH_2Cl_2, and washed with H_2O three times. The organic layer was separated, dried, and concentrated. The mixture was then purified by silica column chromatography (MeOH : CH_2Cl_2 = 1:20) to give **C3** as a red powder (55 mg, 59.7 µmol, 50% yield). **^{1}H NMR (400 MHz, $CDCl_3$):** δ (ppm): 13.96 (s, 1H), 13.23 (s, 1H), 8.87-8.80 (m, 1H), 8.06-7.99 (m, 1H), 7.78 (td, J = 8.0, 2.8 Hz, 1H), 7.39 (d, J = 8.4 Hz, 1H), 7.28 (dd, J = 5.2, 2.0 Hz, 1H), 6.90 (dd, J = 14.0, 5.2 Hz, 1H), 6.18 (d, J = 4.0 Hz, 1H), 5.75-5.72 (m, 1H), 5.59-5.56 (m, 1H), 5.49 (d, J = 3.6 Hz, 1H), 5.30-5.22 (m, 2H), 4.92-4.82 (m, 1H), 4.75 (s, 2H), 4.72-4.65 (dd, J = 12.4, 2.4 Hz, 1H), 4.44-4.16 (m, 7H), 4.07 (d, J = 3.2 Hz, 3H), 3.87-3.78 (m, 1H), 3.68-3.57 (m, 4H), 3.26 (d, J = 18.8 Hz, 1H), 3.00 (d, J = 18.8 Hz, 1H), 2.33 (d, J = 14.8 Hz, 1H), 2.17 (dd, J = 14.8, 4.0 Hz, 1H), 1.98-1.90 (m, 5H), 1.37-1.28 (m, 6H). **^{13}C NMR (101 MHz, $CDCl_3$):** δ (ppm): 213.94, 187.21, 186.77, 167.28, 164.71, 162.14, 161.99, 161.16, 156.33, 155.76, 155.05, 146.30, 145.70, 141.27, 136.11, 135.96, 135.58, 133.79, 133.68, 126.19, 120.92, 119.99, 118.58, 111.67, 111.51, 100.69, 95.76, 84.92, 69.64, 69.40, 67.38, 65.67, 63.27, 62.50, 62.30, 56.81, 47.23, 38.66, 35.74, 34.13, 30.17, 18.39, 16.97, 14.07. **ESI-MS (*m/z*)** for $C_{45}H_{48}N_2O_{19}$ expected $[M+Na]^+$: 943.2743, Found for $[M+Na]^+$: 943.2670.

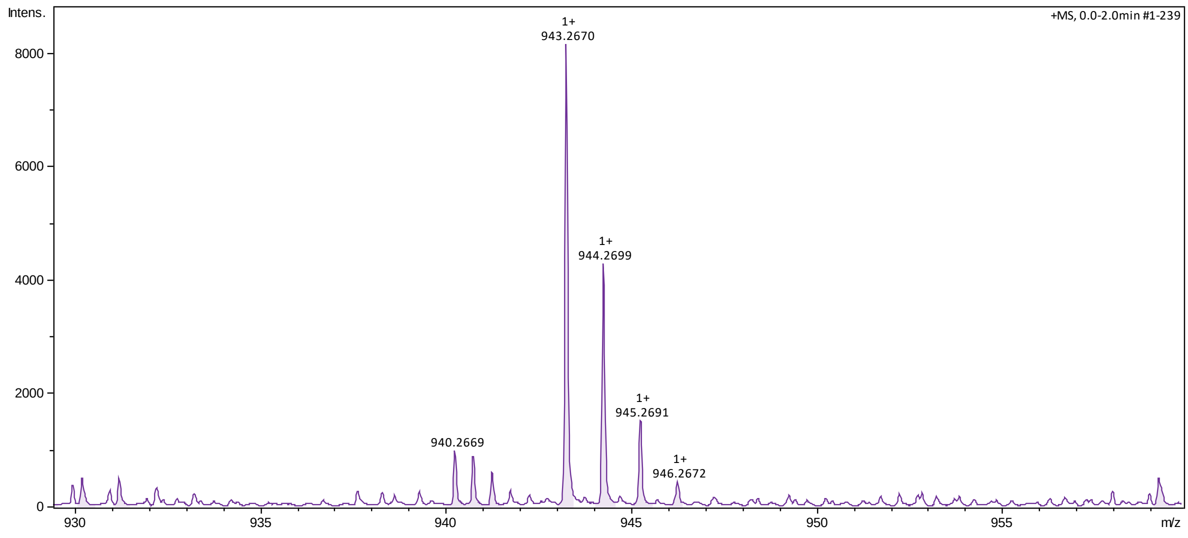

ESI-MS (*m/z*) of Ethyl 1-(((((2S,3R,4S)-3-hydroxy-2-methyl-6-(((1S,3S)-3,5,12-trihydroxy-3-(2-hydroxyacetyl)-10-methoxy-6,11-dioxo-1,2,3,4,6,11-hexahydrotetracen-1-yl)oxy)tetrahydro-2H-pyran-4-yl)carbamoyl)oxy)met-hyl)-3-((2 (methacryloyloxy)ethyl)carbamoyl)-7-oxabicyclo[2.2.1]hepta-2,5-diene-2-carboxylate (**C3**).

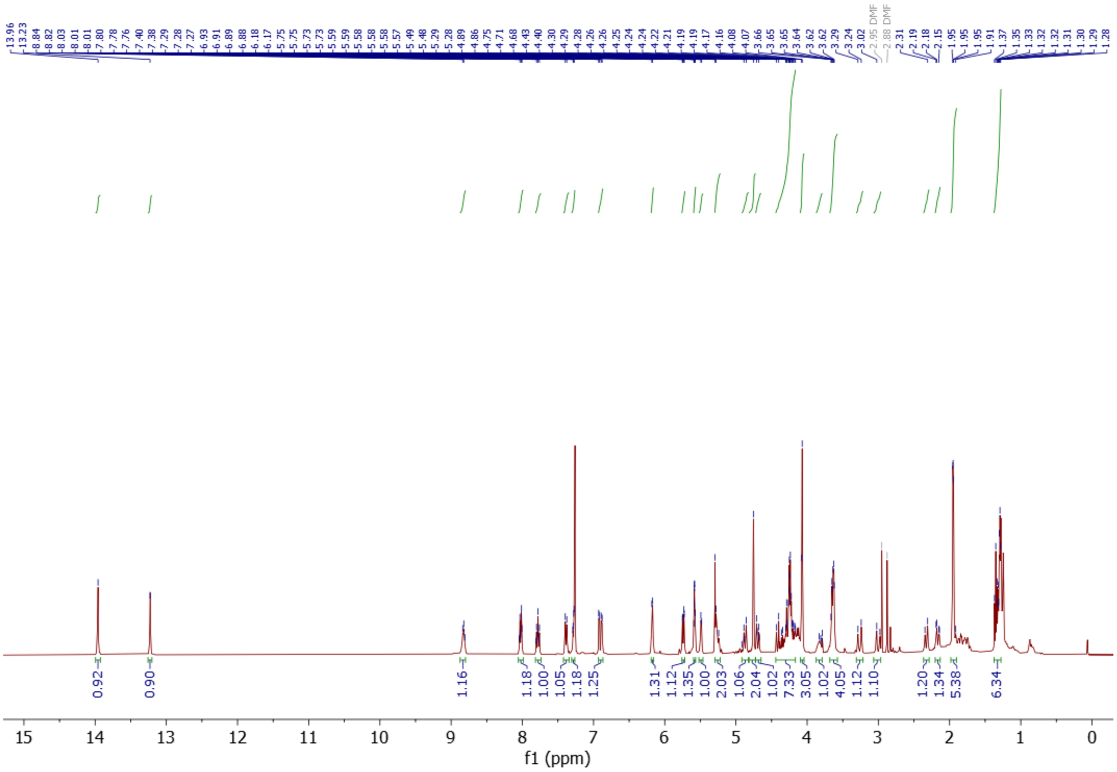

^{1}H NMR spectrum of Ethyl 1-(((((2S,3R,4S)-3-hydroxy-2-methyl-6-(((1S,3S)-3,5,12-trihydroxy-3-(2-hydroxyacetyl)-10-methoxy-6,11-dioxo-1,2,3,4,6,11-hexahydrotetracen-1-yl)oxy)tetrahydro-2H-pyran-4-yl)carbamoyl)oxy)met-hyl)-3-((2 (methacryloyloxy)ethyl)carbamoyl)-7-oxabicyclo[2.2.1]hepta-2,5-diene-2-carboxylate(**C3**).

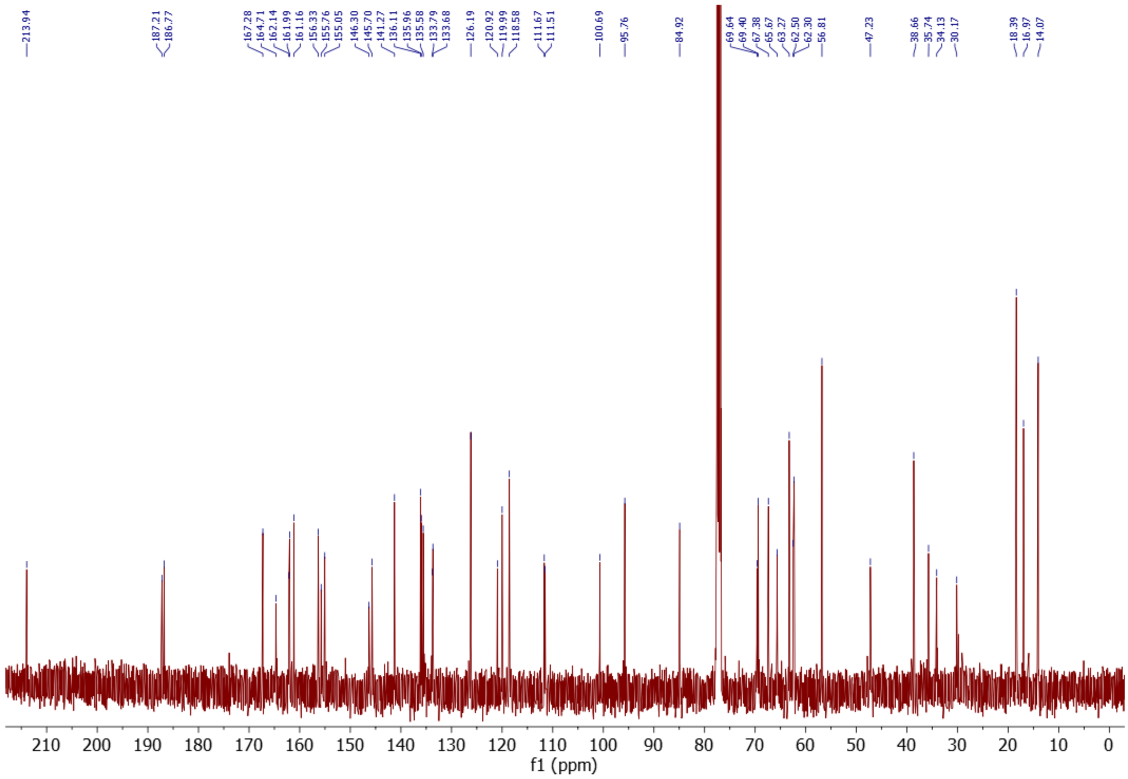

^{13}C NMR spectrum of Ethyl 1-(((((2S,3R,4S)-3-hydroxy-2-methyl-6-(((1S,3S)-3,5,12-trihydroxy-3-(2-hydroxyacetyl)-10-methoxy-6,11-dioxo-1,2,3,4,6,11-hexahydrotetracen-1-yl)oxy)tetrahydro-2H-pyran-4-yl)carbamoyl)oxy)methyl)-3-((2-(methacryloyloxy)ethyl)carbamoyl)-7-oxabicyclo[2.2.1]hepta-2,5-diene-2-carboxylate (**C3**).

2.7. Furan-2-ylmethyl ((2S,3R,4S,6R)-3-hydroxy-2-methyl-6-(((1S,3S)-3,5,12-trihydroxy-3-(2-hydroxyacetyl)-10-methoxy-6,11-dioxo-1,2,3,4,6,11-hexahydrotetracen-1-yl)oxy)tetrahydro-2H-pyran-4-yl)carbamate (**D1**)

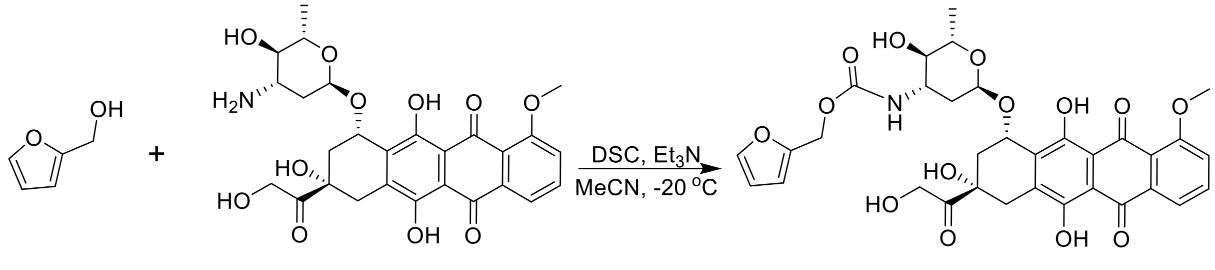

The preparation followed an adapted literature protocol. *N,N'*-disuccinimidyl carbonate (132 mg, 517 µmol, 3.0 equiv.), Et_3N (90 µL, 690 µmol, 4.0 equiv.) were dissolved in MeCN (1 mL) in a round bottom flask. The mixture was cooled to 0 °C and a solution of 2-(Hydroxymethyl)furan (51 mg, 520 µmol, 3.02 equiv.) in MeCN (1 mL) was added. The reaction mixture was allowed to stir in an ice bath for 1 h. Subsequently, the mixture was dried *in vacuo* and used without further purification. The obtained material was dissolved in 1.5 mL CH_2Cl_2 and added dropwise into a cooled (-20 °C) solution of doxorubicin hydrochloride (100 mg, 172 µmol, 1.0 equiv.) and Et_3N (96 µL, 689 µmol, 4.0 equiv.) in DMF (2 mL). The reaction mixture was stirred for 1 h at -20 °C and then warmed up slowly to room temperature over 3 h. Afterwards, the solvent was removed *in vacuo*, the residue redissolved in CH_2Cl_2, and washed with H_2O three times. The organic layer was separated, dried, and concentrated. The mixture was then purified by silica column chromatography (MeOH : CH_2Cl_2 = 1:30) to give **D1** as a red powder (75 mg, 65% yield). **^{1}H NMR (400 MHz, $CDCl_3$):** δ (ppm): 13.89 (s, 1H), 13.13 (s, 1H), 7.99 – 7.95 (m, 1H), 7.74 (t, J = 8.0 Hz, 1H), 7.4-7.3 (m, 2H), 6.36 – 6.23 (m, 2H), 5.47 (d, J = 3.2 Hz, 1H), 5.35 – 5.28 (m, 1H), 5.21 (s, 1H), 4.95 (s, 2H), 4.74 (s, 2H), 4.12 (q, J = 6.4 Hz, 1H), 4.04 (s, 3H), 3.84 (s, 1H), 3.64 (s, 1H), 3.20 (d, J = 18.8, 1H), 2.87 (d, J = 18.8, 1H), 2.30 (d, J = 14.8 Hz, 1H), 2.13 (dd, J = 14.8, 4.0 Hz, 1H), 1.86-1.72 (m, 2H), 1.27 (d, J = 6.4 Hz, 3H). **^{13}C NMR (101 MHz, $CDCl_3$):** δ (ppm): 214.00, 186.98, 186.59, 161.07, 156.27, 155.61, 155.38, 149.91, 143.26, 135.85, 135.44, 133.71, 133.68, 120.78, 119.89, 118.56, 111.56, 111.39, 110.61, 110.51, 100.83, 76.68, 69.69, 69.56, 67.45, 65.64, 58.60, 56.72, 47.15, 35.69, 33.98, 30.17, 16.93.

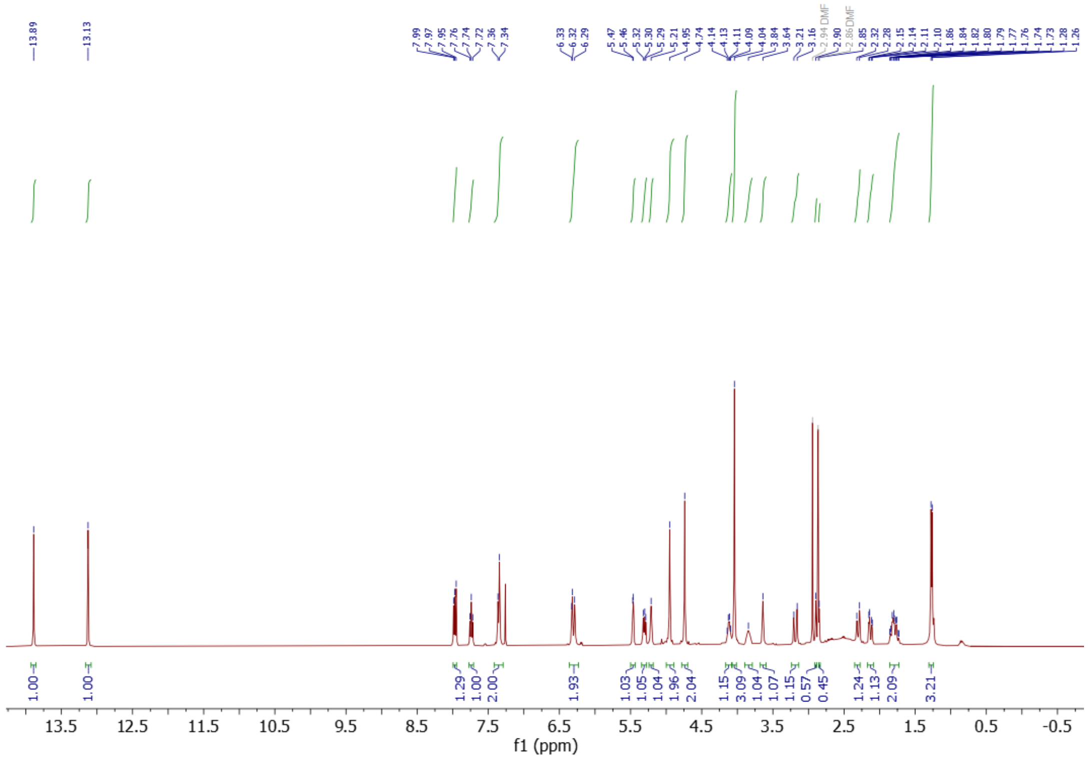

^{1}H NMR spectrum of furan-2-ylmethyl ((2S,3R,4S,6R)-3-hydroxy-2-methyl-6-(((1S,3S)-3,5,12-trihydroxy-3-(2-hydroxyacetyl)-10-methoxy-6,11-dioxo-1,2,3,4,6,11-hexahydrotetracen-1-yl)oxy)tetrahydro-2H-pyran-4-yl)carbamate (**D1**).

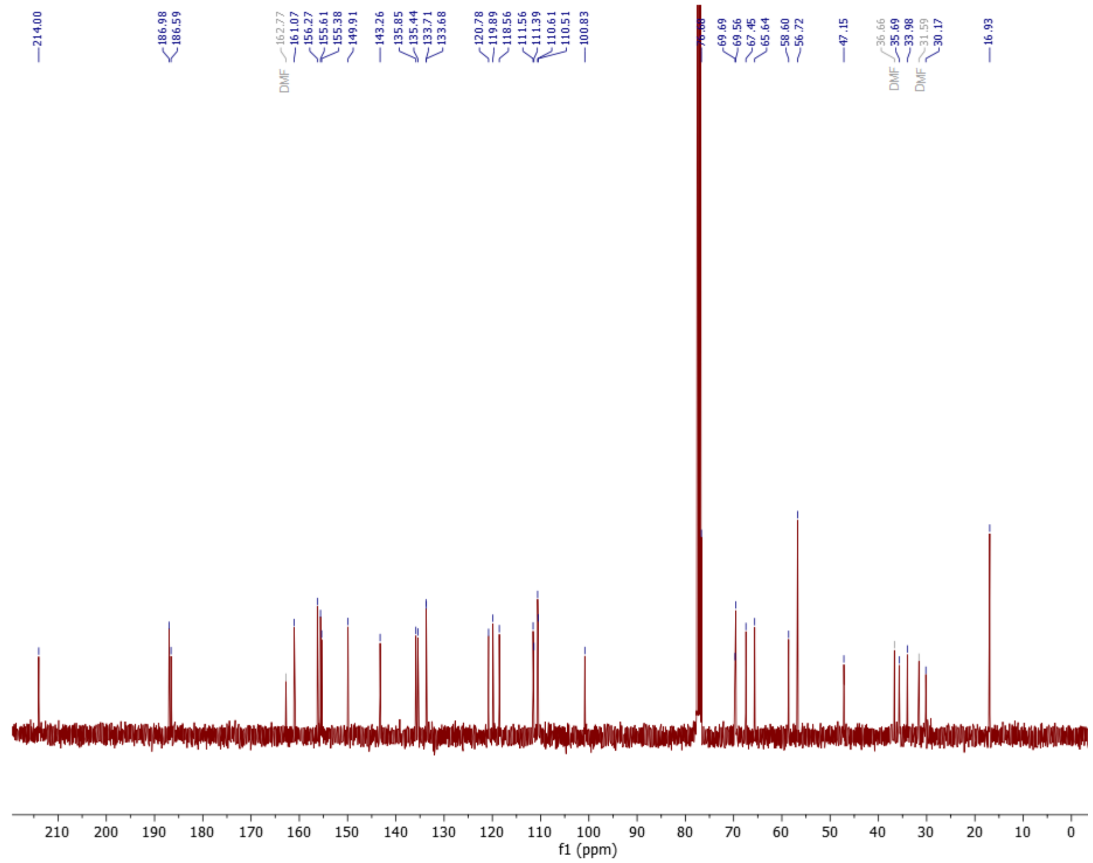

^{13}C NMR spectrum of furan-2-ylmethyl ((2S,3R,4S,6R)-3-hydroxy-2-methyl-6-(((1S,3S)-3,5,12-trihydroxy-3-(2-hydroxyacetyl)-10-methoxy-6,11-dioxo-1,2,3,4,6,11-hexahydrotetracen-1-yl)oxy)tetrahydro-2H-pyran-4-yl)carbamate (**D1**).

3. Sonication Systems

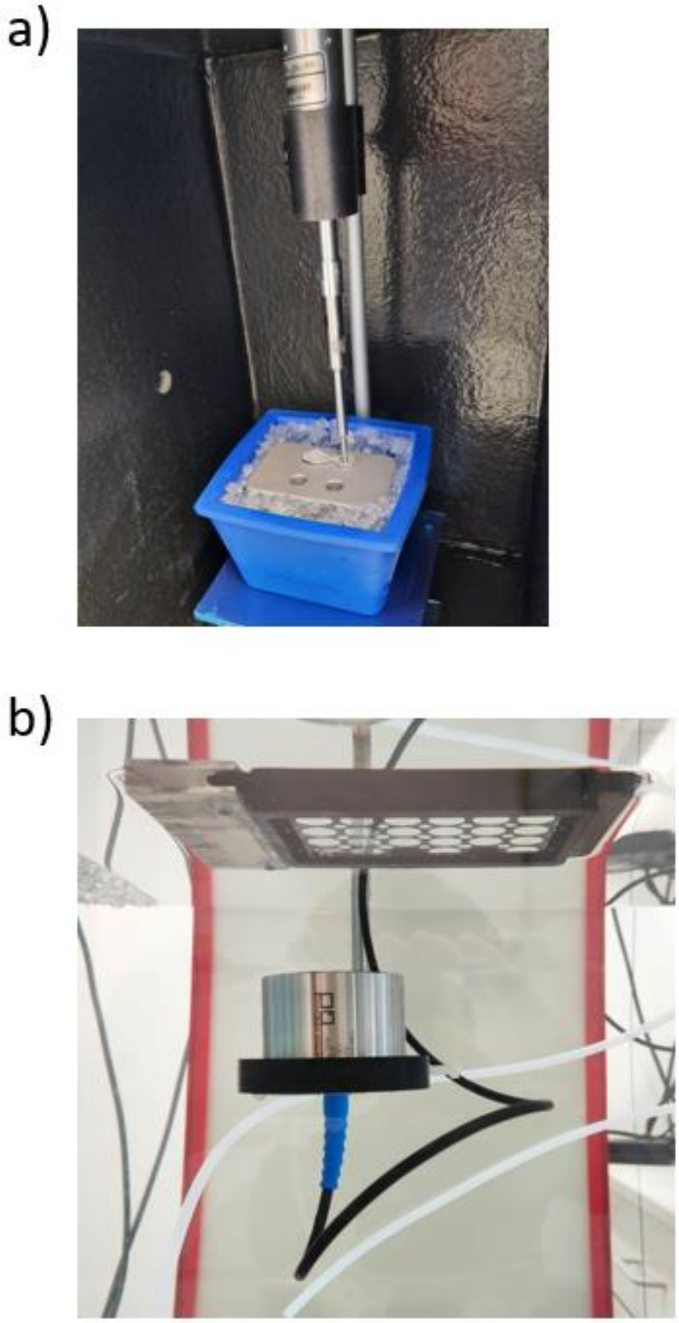

Figure S1. Sonication system. (**a**) 20 kHz sonicator. (**b**) 1.5 MHz HIFU transducer.

4. Synthesis and Analysis of Polymers

4.1. Synthesis of Linear Polymers (LPs)

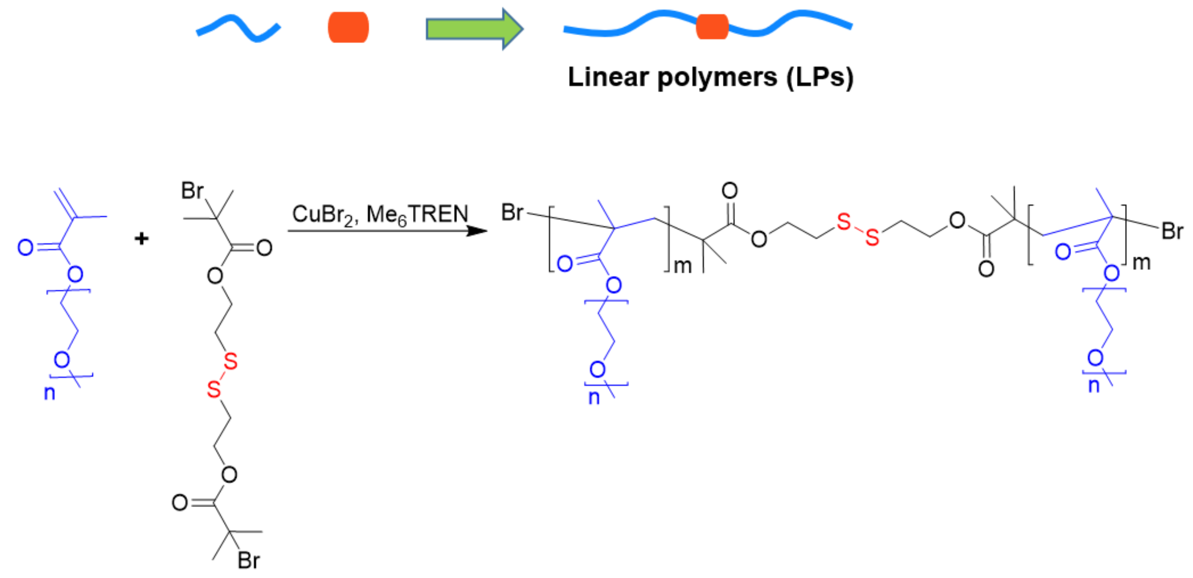

Scheme S2: Synthesis of LPs.

Disulfide mechanophore contained linear polymer (LPs) were prepared according reported literature.[6] LPs was synthesized as follows. PEGMEMA (4.0 g, 13.3 mmol, 121 equiv.), Bis[2 (2' bromoisobutyryloxy)ethyl] disulfide (50 mg, 0.11 mmol, 1.0 equiv.), Me_6TREN (0.55 mg, 2.4 μmol) and $CuBr_2$ (0.29 mg, 1.3 μmol) were dissolved in DMSO (3 mL) in a Schlenk flask and sealed with a rubber septum reinforced with a cable tie. The solution was degassed by 3 consecutive freeze-pump-thaw cycles. During that time, copper wire (6 cm) was activated in 37% HCl for 30 min, subsequently washed with H_2O and acetone, and then dried. The copper wire was added to the reaction solution and then polymerization was allowed to proceed for 6 h ($M_n \approx$ 47 kDa) at room temperature. The viscous solution was diluted with THF to terminate the polymerization by passing through a plug of basic Al_2O_3. After concentration *in vacuo*, THF mixture was added dropwise to ice-cold Et_2O under stirring. Et_2O was decanted and the viscous polymer redissolved in THF, it was again precipitated in fresh Et_2O. Disulfide centered linear polymers with molar masses of 47 kDa was obtained after repeating the precipitation process for three times.

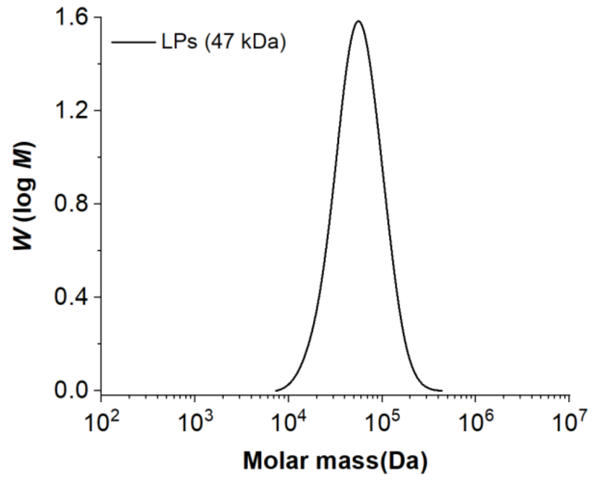

Figure S2. GPC RI molar mass distributions of LPs.

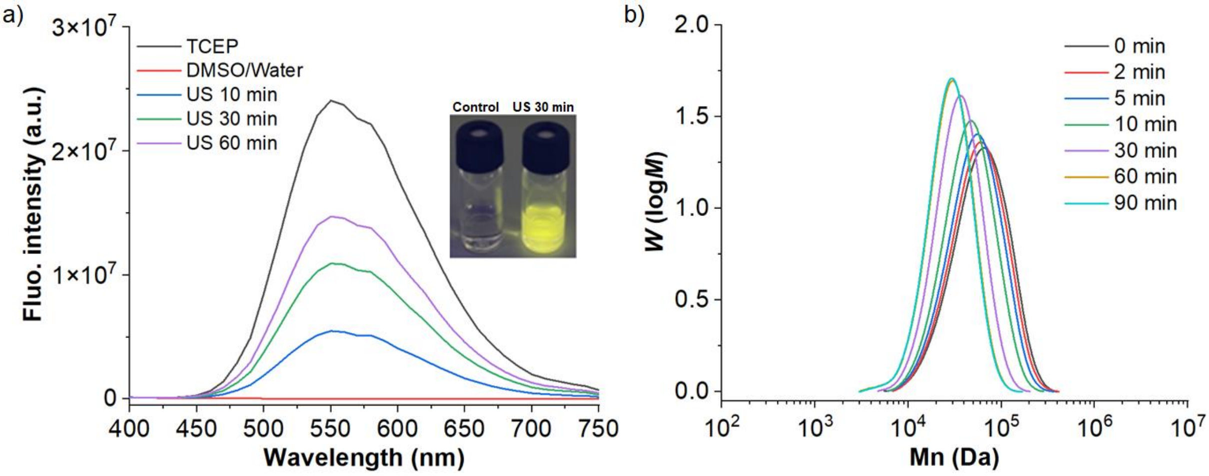

Figure S3. (**a**) Fluorescence spectra of the mixture of LPs and OND sensor, before and after 20 kHz (I = 12.4 W cm^{-2}) sonication (Solvent: DMSO/H_2O). Inset: photograph of the solutions under UV-light (λ_{exc} = 365 nm). (**b**) M_n of LPs with different sonication time (20 kHz, I = 12.4 W cm^{-2}).

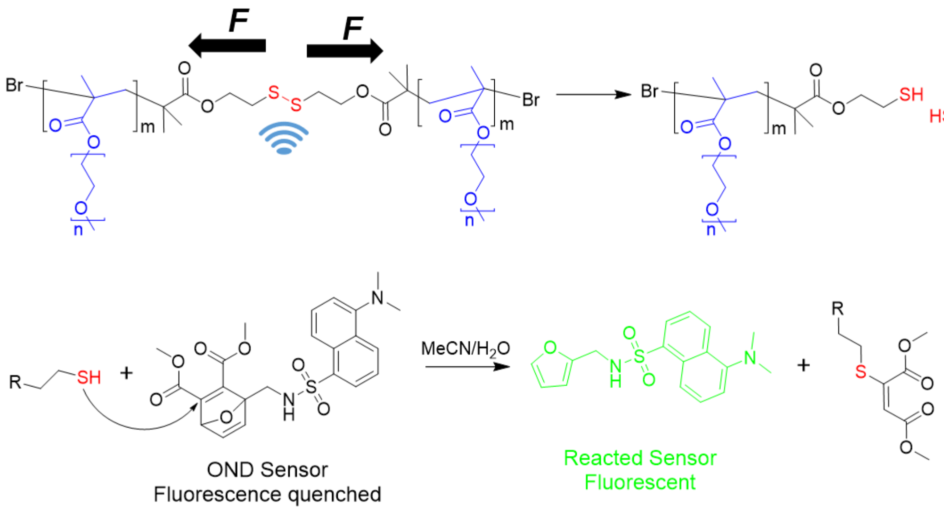

Figure S4. Disulfide mechanophore activation and the mechanism of thiols react with OND sensor.

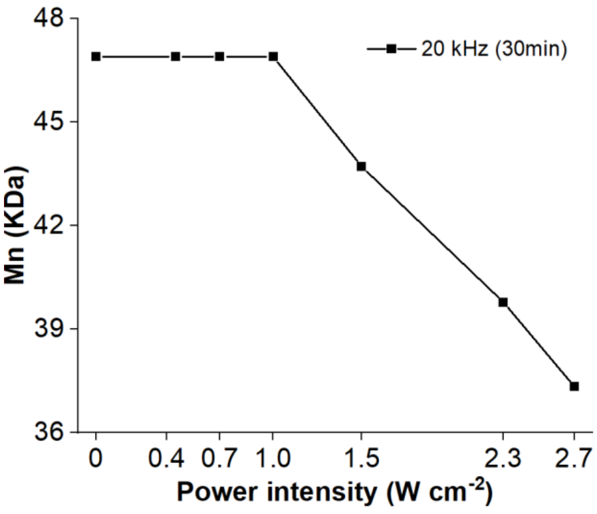

Figure S5. The M_n changes of LPs with the US I from 0 W cm^{-2} to 2.7 W cm^{-2}.

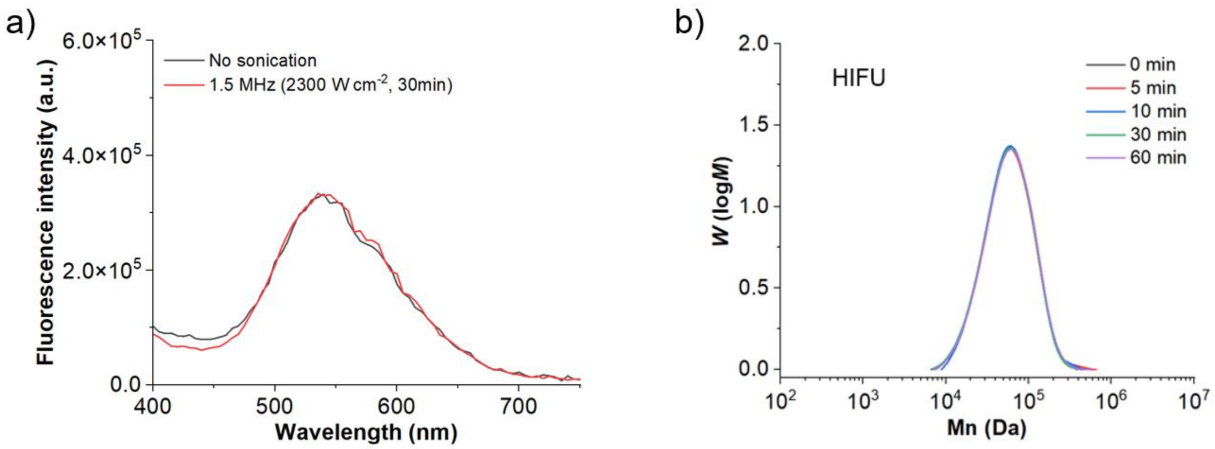

Figure S6. (**a**) Fluorescence spectra of the mixture of **B2** and LPs, after 1.5 MHz (32 W, I = 2300 W cm^{-2}) HIFU sonication. (**b**) GPC RI molar mass distributions of LPs after 1.5 MHz (32 W, I = 2300 W cm^{-2}) HIFU sonication.

4.2. Synthesis of Network Core-Structured Star Polymers (NCSPs)

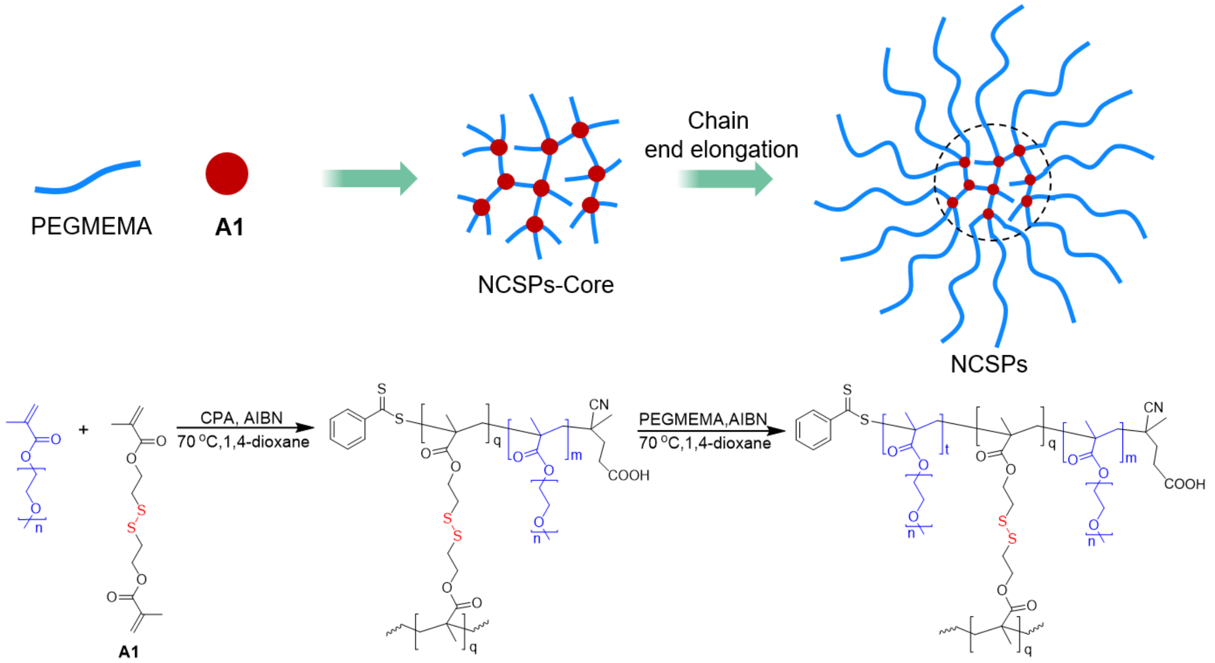

Scheme S3: Synthesis of NCSPs.

Network core-structured star polymers (NCSPs) were prepared according to reported literature.[7]

(1) Synthesis of NCSPs-core: The Network core-structured star polymers core was synthesized as follows. PEGMEMA (850 mg, 2.83 mmol, 14.1 equiv.), **A1** (58 mg, 0.2 mmol, 1.0 equiv.) were dissolved in 1,4-dioxane (3 mL) with CPA RAFT agent (29 mg, 0.1 mmol) and AIBN (4.3 mg, 0.026 mmol) in a reaction flask and sealed with a rubber septum reinforced with a cable tie. The solution was degassed by 3 consecutive freeze-pump-thaw cycles and then stirred at 70 °C for 24 h. After 24 h of the polymerization reaction, the solution was directly placed at -20 °C for approximately 20 min and then exposed to air for termination of the polymerization. Then, 5 mL THF was added to the mixture, after concentration *in vacuo*, the mixture was added dropwise to stirred ice-cold Et_2O. Et_2O was decanted and the viscous polymer redissolved in THF, it was again precipitated in fresh Et_2O. After repeating the precipitation process three times, the unreacted monomer and impurities removed by dialysis for 48 h.

(2) Synthesis of NCSPs: Network core-structured star polymers were synthesized by chain extension of a NCSPs -core with PEGMEMA. Briefly, NCSPs cores (50 mg) were dissolved in 1,4-dioxane (1 mL) with PEGMEMA

(645 mg, 2.15 mmol, 10.7 equiv.) and AIBN (1.7 mg, 0.01 mmol) in a reaction flask and sealed with a rubber septum reinforced with a cable tie. The solution was degassed by 3 consecutive freeze-pump-thaw cycles and then stirred at 70 °C for 6 h, then 600 mg NCSPs was obtained.

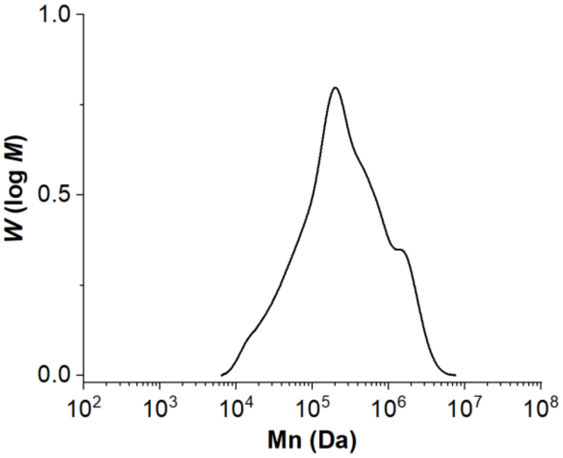

Figure S7. GPC RI molar mass distribution of NCSPs (M_n ~109 kDa).

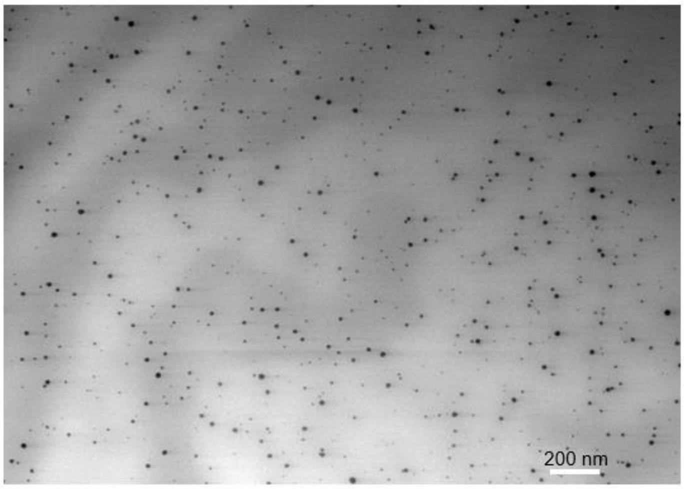

Figure S8. TEM image of NCSPs.

(3) The structure of NCSPs: The structure of NCSPs was defined by GPC and ^{1}H NMR, the arms number and monomers number of an arm were calculated. First, the ratio of CPA and PEGMEMA in the core structure was calculated by ^{1}H-NMR with the following equation:[7c]

$$\boldsymbol{R} = \frac{I^{4.0\ \mathrm{ppm}}}{2 \cdot I^{7.5\ \mathrm{ppm}}}$$

where $\mathbf{I}^{7.5\ \mathrm{ppm}}$ corresponded to signal intensities at 7.5 ppm attributed to RAFT agent CPA (see **Figure S9a**) and where $\mathbf{I}^{4.0\ \mathrm{ppm}}$ corresponded to signal intensities at 4.0 ppm attributed to **A1** (crosslinker) and PEGMEMA. Due to **A1** (290 Da) and PEGMEMA (M_n = 300 Da) having comparable molar mass, here we merge the two molecules and treat them as one molecule as PEGMEMA.

Then, we obtain the ratio (**R**) of PEGMEMA (300 Da) and CPA (279 Da) is 28:2 (14:1) (see **Figure S9b**). In addition, the GPC result show that the molar mass (M_n) of the core structure of star polymers is around 31000 Da (see **Figure 10**).

After our calculation:

M_{core} = 31000 Da ≈ (300·14+279·1)·**7**

Therefore, we extracted that the core structure contained ~7 CPA molecules, which means the core structure on average had 7 arms. In the next step of star polymers synthesis, PEGMEMA monomers are continuously inserted at the RAFT end to achieve chain elongation (see **Figure S11**).

Then, the average molar mass of an arm was calculated:

$M_{arm} = (M_{total} – M_{core})/7$ = (108500–31000)/7 = 11071 Da

Next, the average arm length (degree of polymerization) of an arm was calculated:

$X_n = M_{arm}/M_{monomer}$ = 11071/300 ≈ 37.

Then, we know that one arm contained 37 PEGMEMA monomers.

In addition, the "spanning molar mass" M_{span} of star polymers and linear polymers were calculated, defined as:[7d]

For NCSPs: $M_{span} = 2M_{arm} + M_{core}$

where M_{core} is the molar mass of the core structure. We calculated the M_{span} ≈ 54 kDa.

For linear polymers (LPs) with the molar mass of 47 kDa, $M_{span} = M$ = 47 kDa.

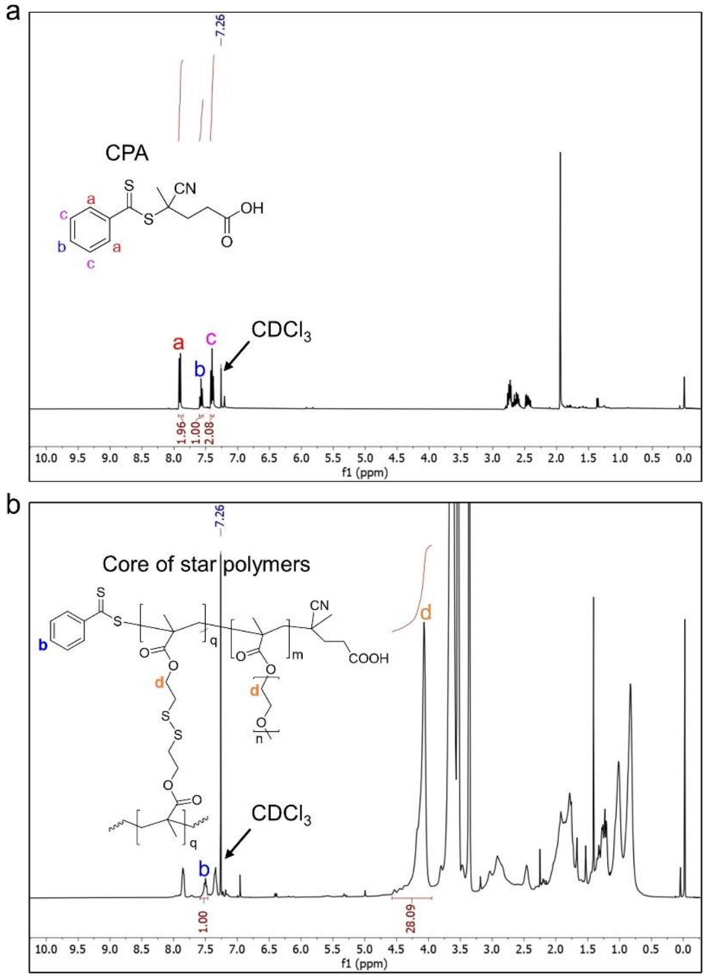

Figure S9. ^{1}H-NMR spectra of RAFT agent (CPA) and core of star polymers recorded in $CDCl_3$ at 20 °C.

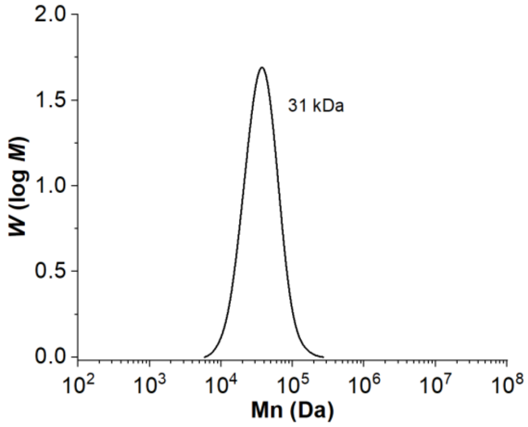

Figure S10. GPC result of core structure of star polymers.

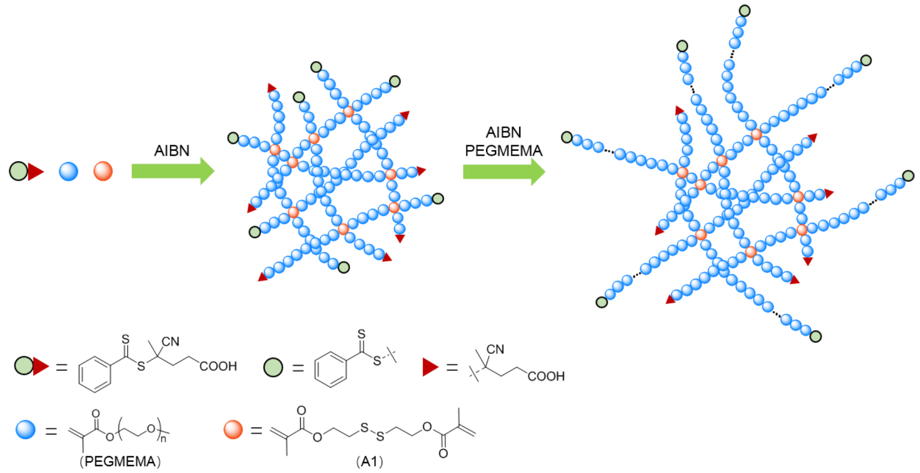

Figure S11. The synthesis process of NCSPs.

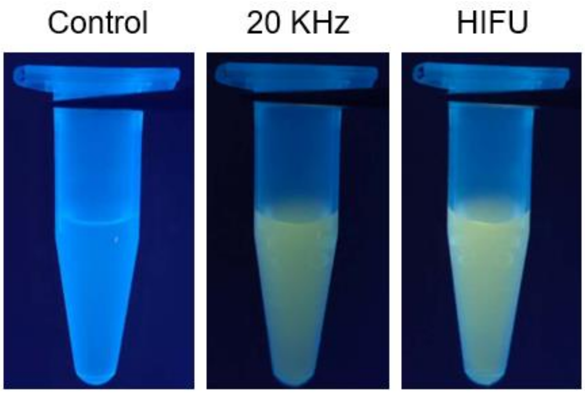

Figure S12. Fluorescence of the mixture of **B2** and NCSPs, after 30 min sonication. 20 kHz (I = 12.4 W cm^{-2}), 1.5 MHz (32 W, I = 2300 W cm^{-2}).

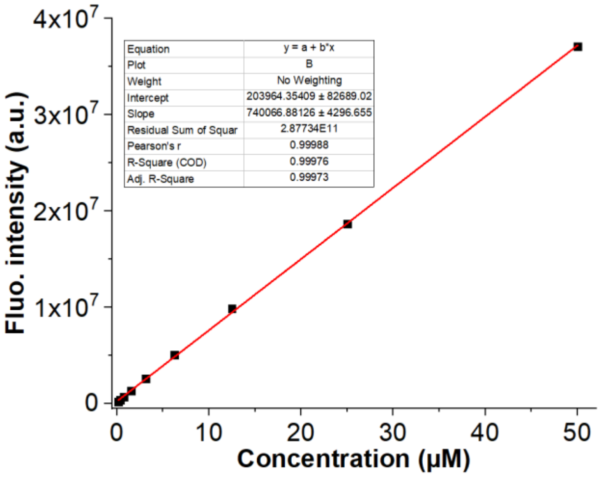

Figure S13. The standard curve plotted by **B1** concentration and fluorescence intensity.

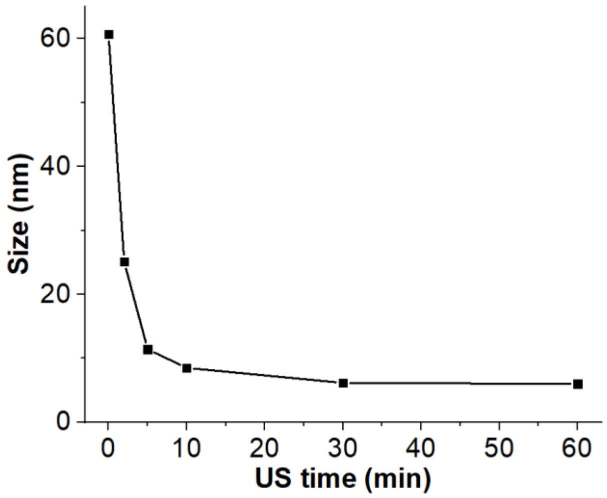

Figure S14. Size change of NCSPs after sonication (20 kHz, I = 12.4 W cm^{-2}).

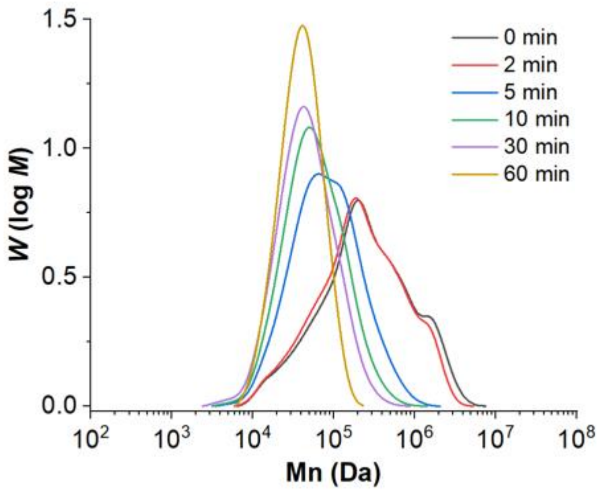

Figure S15. GPC RI molar mass distributions of NCSPs after different times of HIFU sonication (32 W, I = 2300 W cm^{-2}).

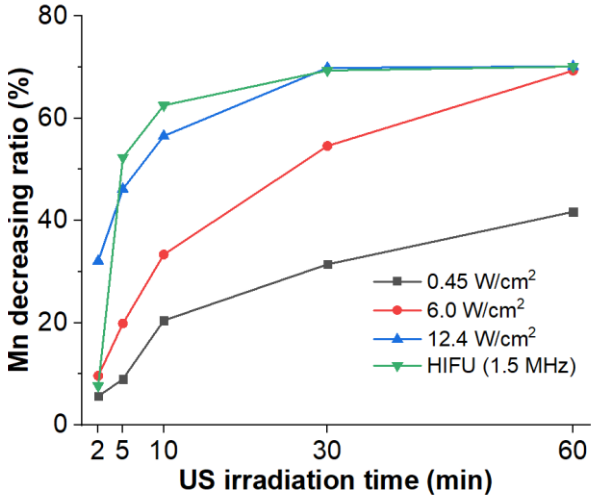

Figure S16. M_n decreasing ratio of NCSPs with different sonication intensity.

4.3. Synthesis of Mechanophore-free Network Core-Structured Star Polymers (FNCSPs)

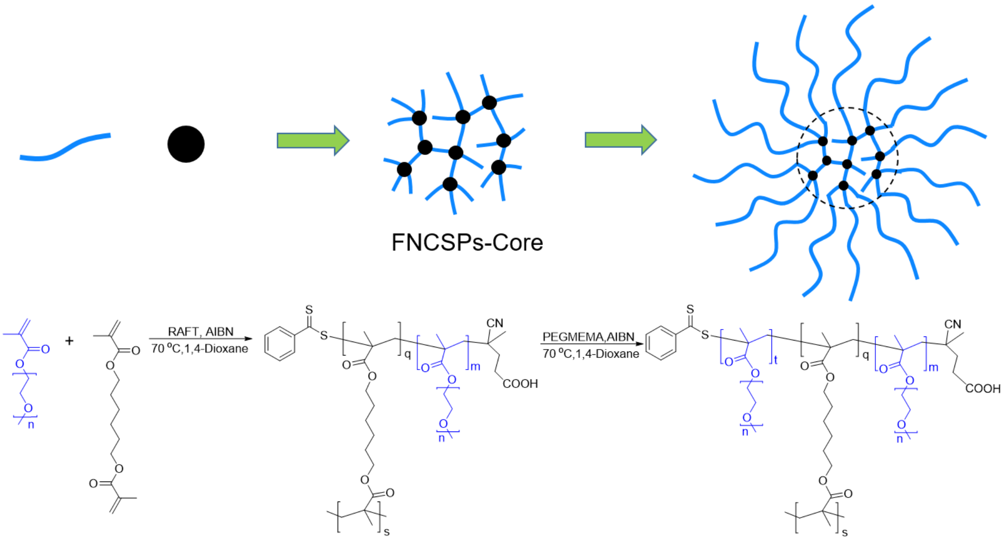

Scheme S4: Synthesis of FNCSPs.

(1) Synthesis of FNCSPs-core: The FNCSP core was synthesized as follows. PEGMEMA (850 mg, 2.83 mmol, 14.1 equiv.), 1,6-hexanediol dimethacrylate (51 mg, 0.2 mmol, 1.0 equiv.) were dissolved in 1,4-dioxane (3 mL) with CPA RAFT agent (29 mg, 0.1 mmol) and AIBN (4.3 mg, 0.026 mmol) in a reaction flask and sealed with a rubber septum reinforced with a cable tie. The solution was degassed by 3 consecutive freeze-pump-thaw cycles and then stirred at 70 °C for 24 h. After 24 h of the polymerization reaction, the solution was directly placed at -20 °C for approximately 20 min and then exposed to air for termination of the polymerization. Then, 5 mL THF was added to the mixture, after concentration *in vacuo*, the mixture was added dropwise to stirred ice-cold Et_2O. Et_2O was decanted and the viscous polymer redissolved in THF, it was again precipitated in fresh Et_2O. After repeating the precipitation process three times, the unreacted monomer and impurities removed by dialysis for 48 h.

(2) Synthesis of FNCSPs: Non-mechanophore contained network core-structured star polymers were synthesized by chain extension of a FNCSP-core with PEGMEMA. Briefly, FNCSP-core (50 mg) was dissolved in 1,4-dioxane (1 mL) with PEGMEMA (645 mg, 2.15 mmol, 10.7 equiv.) and AIBN (1.7 mg, 0.01 mmol) in a reaction flask and sealed with a rubber septum reinforced with a cable tie. The solution was degassed by 3 consecutive freeze-pump-thaw cycles and then stirred at 70 °C for 6 h, then FNCSPs was obtained.

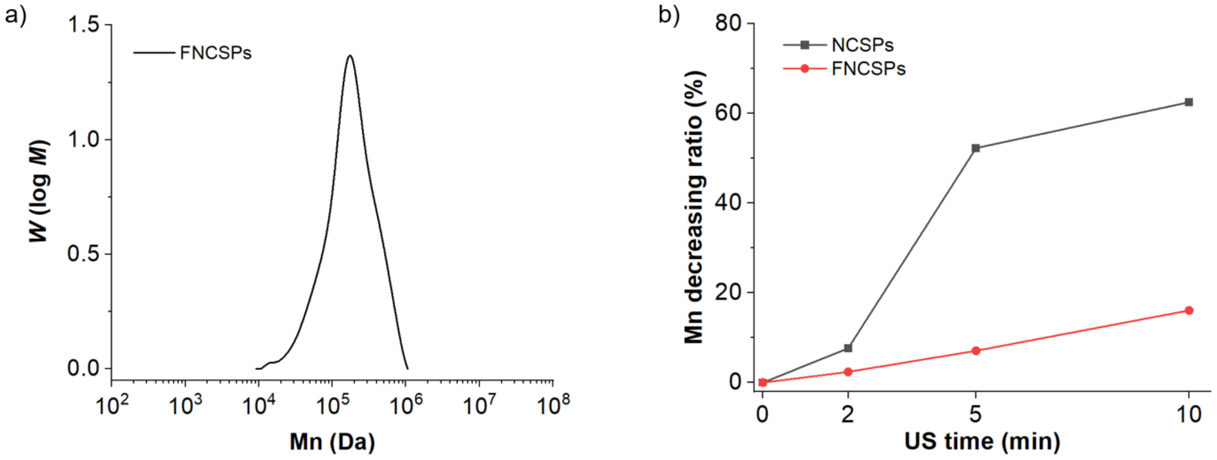

Figure S17. (**a**) GPC RI molar mass distributions of FNCSPs (M_n ≈ 125 kDa). (**b**) M_n decreasing ratio in 10 min with HIFU irradiation (32 W, I = 2300 W cm^{-2}).

4.4. Synthesis of Drug-Containing Network Core-Structured Star Polymers (NCSPs)

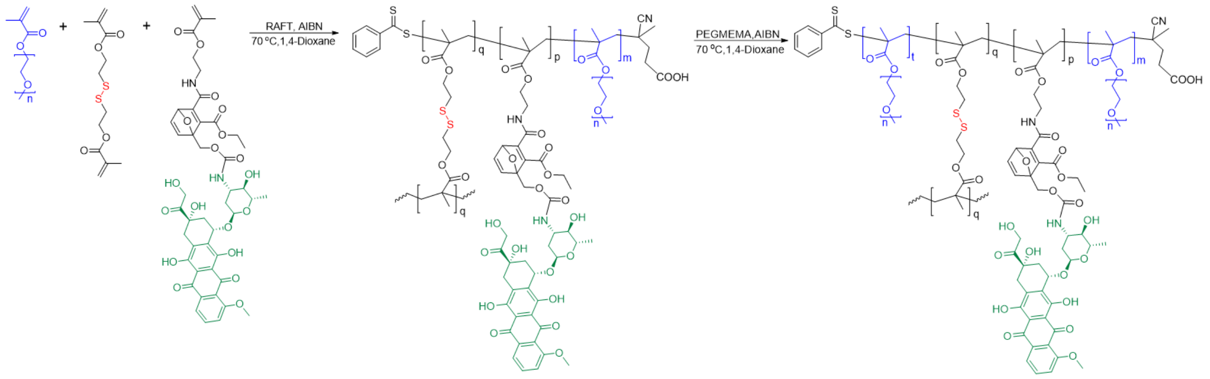

Scheme S5: Synthesis of NCSP-Dox.

Drug contained network core-structured star polymers were prepared according reported literature.[7]

(1) Synthesis of NCSP-core: The NCSP-core copolymer was synthesized as follows. PEGMEMA (850 mg, 2.83 mmol, 70.7 equiv.), **C3** (46 mg, 0.04 mmol, 1.0 equiv.) were dissolved in 1,4-dioxane (3 mL) with CPA RAFT agent (29 mg, 0.1 mmol), **A1** (58 mg, 0.2 mmol, 5.0 equiv.) and AIBN (4.3 mg, 0.026 mmol) in a reaction flask and sealed with a rubber septum reinforced with a cable tie. The solution was degassed by 3 consecutive freeze-pump-thaw cycles and then stirred at 70 °C for 24 h. After 24 h of the polymerization reaction, the solution was directly placed at -20 °C for approximately 20 min and then exposed to air for termination of the polymerization. Then, 5 mL THF was added to the mixture, after concentration *in vacuo*, the mixture was added dropwise to stirred ice-cold Et_2O. Et_2O was decanted and the viscous polymer redissolved in THF, it was again precipitated in fresh Et_2O. After repeating the precipitation process three times, the unreacted monomer and impurities removed by dialysis for 48 h.

(2) Synthesis of NCSP-Dox: Drug contained network core-structured star polymers were synthesized by chain extension of a core copolymer with PEGMEMA. Briefly, NCSP-core (50 mg) was dissolved in 1,4-dioxane (1 mL) with PEGMEMA (645 mg, 2.15 mmol, 53.7 equiv.) and AIBN (1.7 mg, 0.01 mmol) in a reaction flask and sealed with a rubber septum reinforced with a cable tie. The solution was degassed by 3 consecutive freeze-pump-thaw cycles and then stirred at 70 °C for 6 h.

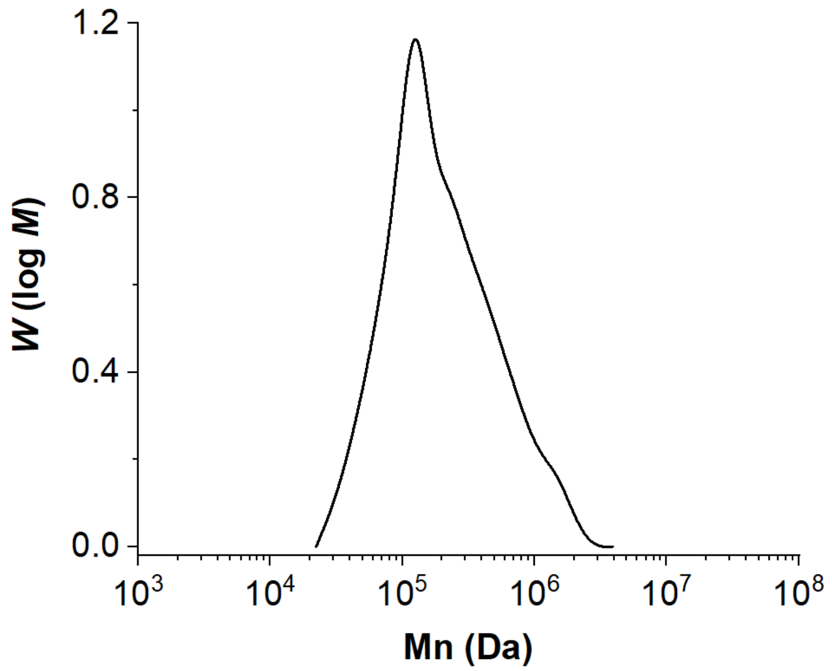

Figure S18. GPC RI molar mass distribution of NCSP-Dox ($M_n \approx$ 133 kDa).

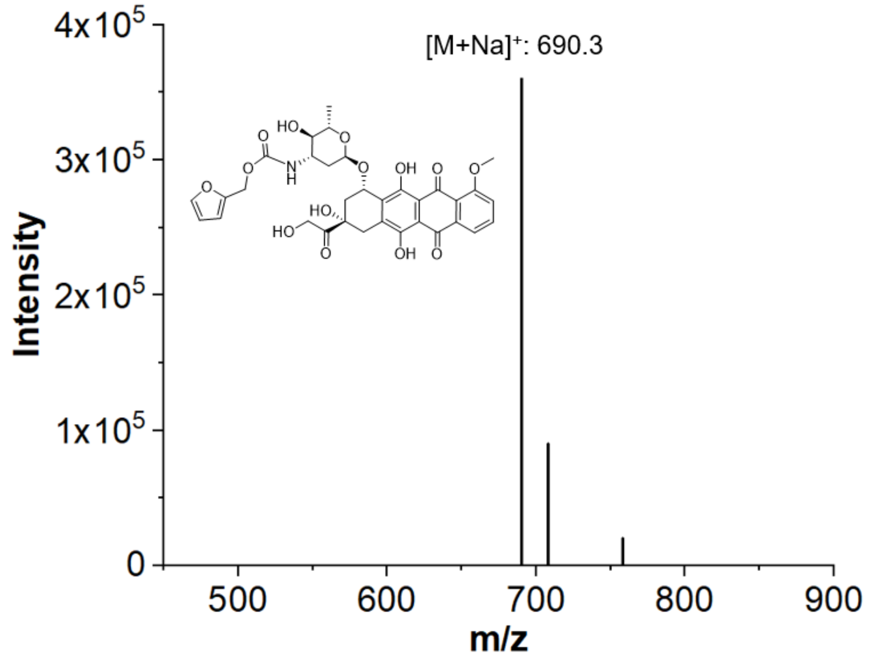

Figure S19. Molar mass of released molecule.

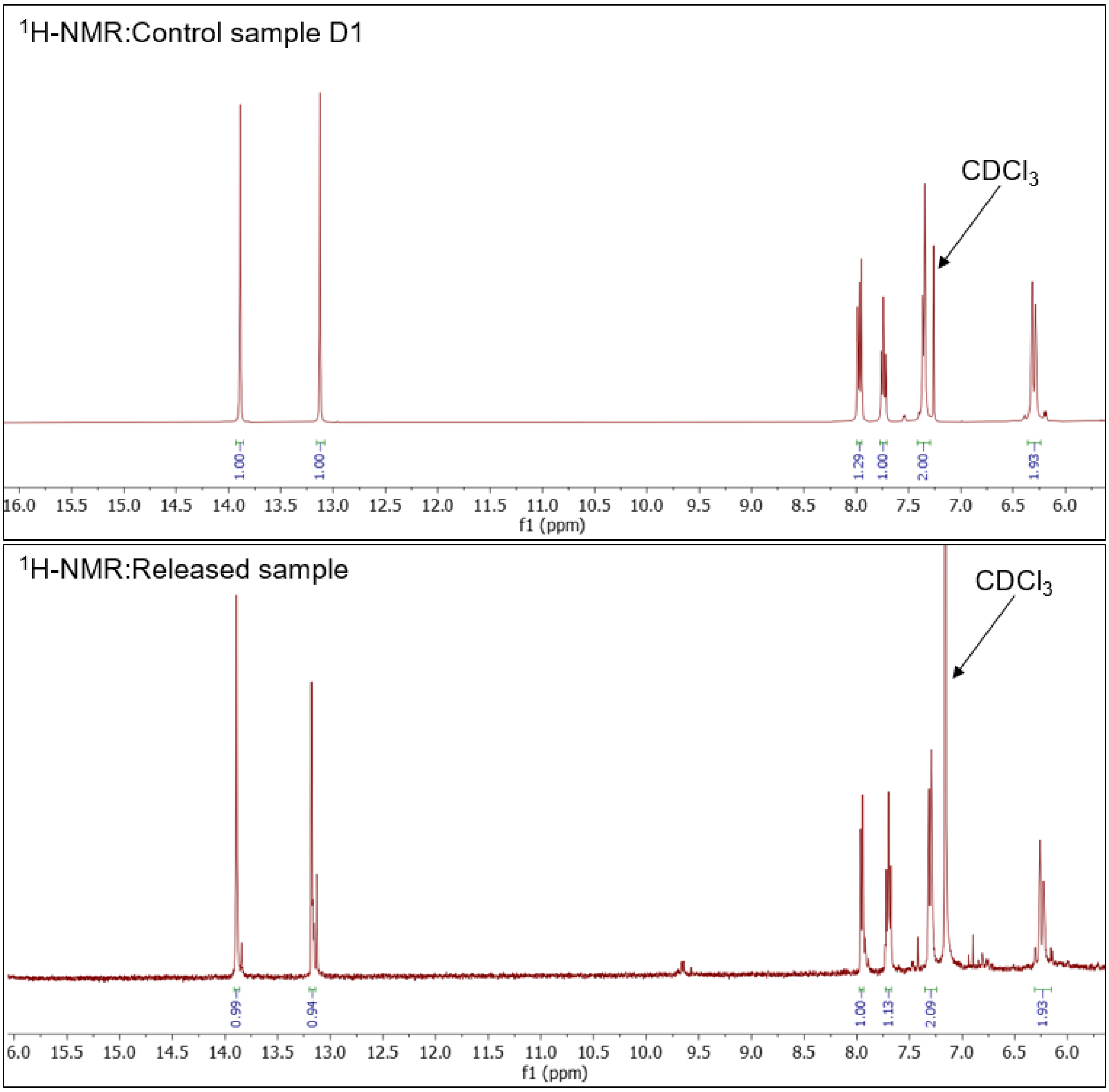

Figure S20. ^{1}H NMR of **D1** and released molecule.

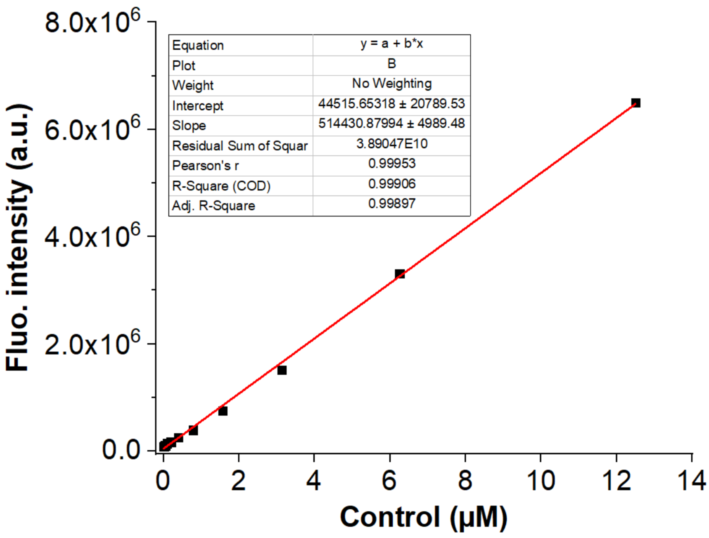

Figure S21. Furan-Dox standard curve (ex: 485 nm, em: 595 nm).

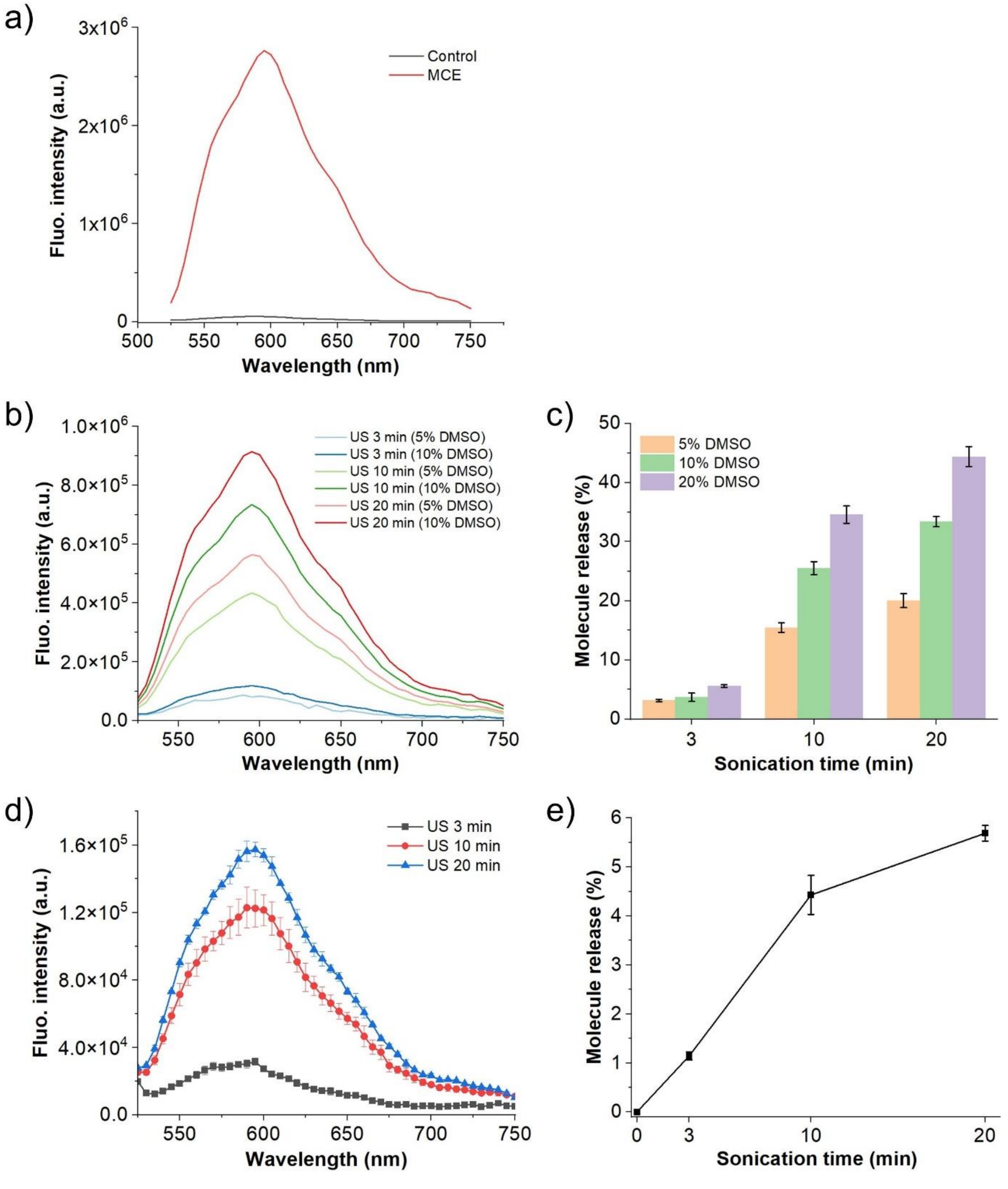

Figure S22. (**a**) Fluorescence spectra of NCSP-Dox treated with excessive MCE (Solvent: DMSO/H_2O). (**b**) Fluorescence spectra (λ_{exc} = 485 nm) of NCSPs after HIFU irradiations (Solvent: 5% and 10% DMSO, 1.5 MHz, I = 2300 W cm^{-2}). (**c**) The release profile of **D1** (Furan-Dox) from NCSPs over sonication time (Solvent: 5% and 10% DMSO, 1.5 MHz, I = 2300 W cm^{-2}). Mean ± SD from the mean. N = 3 independent sonications. (**d**) Fluorescence spectra (λ_{exc} = 485 nm) of NCSPs after HIFU irradiations (Solvent: water, 1.5 MHz, I = 2300 W cm^{-2}). (**e**) The release profile of **D1** (Furan-Dox) from NCSPs over sonication time (Solvent: water, 1.5 MHz, I = 2300 W cm^{-2}). Mean ± SD from the mean. N = 3 independent sonications.

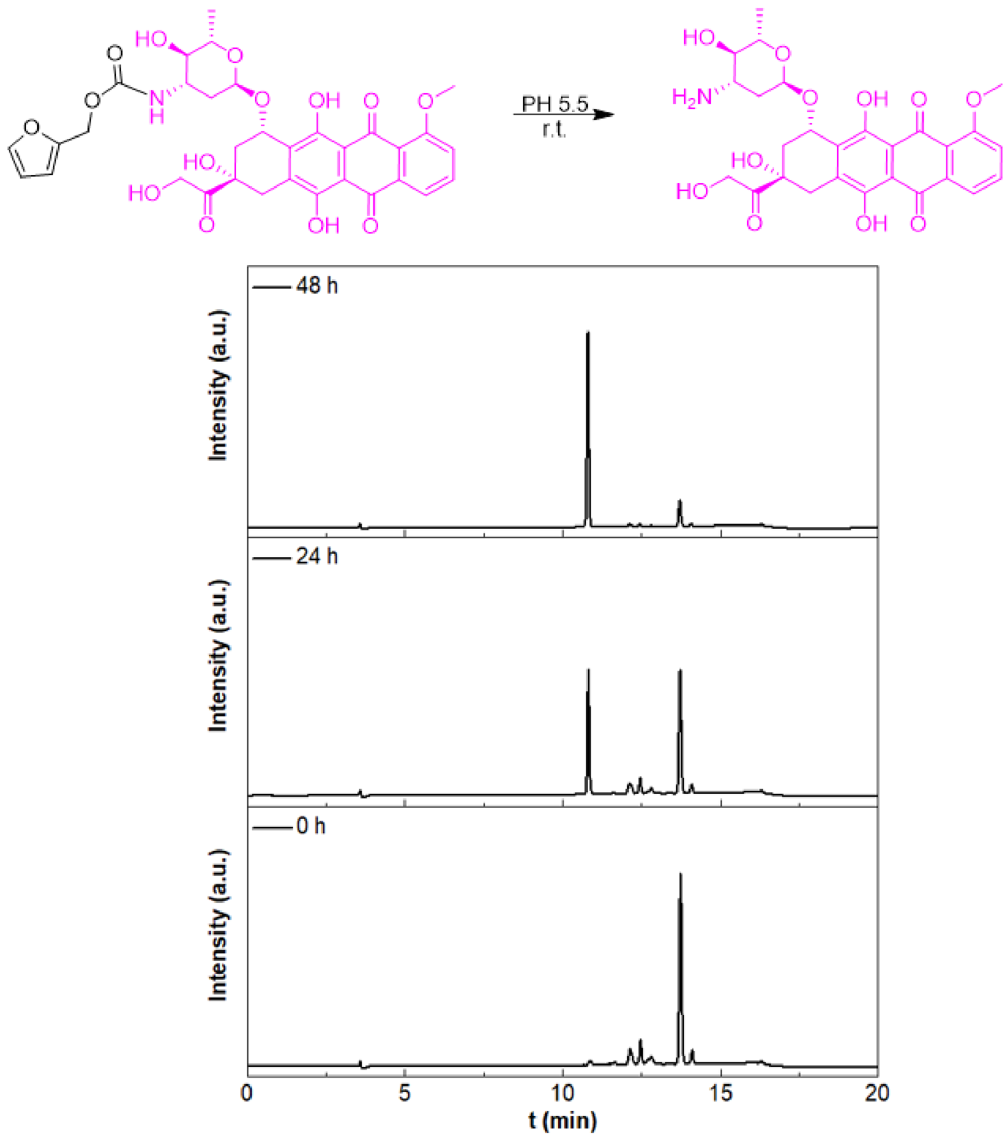

Figure S23. Release kinetics of Dox from released furan-Dox. Released sample was stirred in a mixture of DMSO and PBS buffer at pH = 5.5 for different times at rt, then subjected to UPLC analysis.

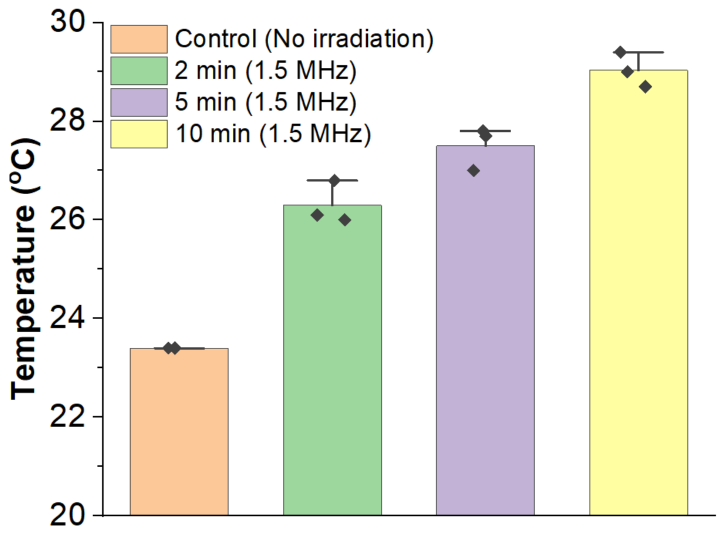

Figure S24. Temperature changes of samples under HIFU irradiation (1.5 MHz, 290 W cm^{-2}, 2, 5, and 10 min).

5. Supplementary Tables

Table S1. The US intensities corresponding to the employed ultrasonic amplitudes for 20 kHz sonications.

Ultrasonic amplitude	25%	30%	35%	40%	45%	50%	75%	100%
Sound Intensity (*I*)	0.45 W/cm^2	0.7 W/cm^2	1.0 W/cm^2	1.5 W/cm^2	2.3 W/cm^2	2.7 W/cm^2	6.0 W/cm^2	12.4 W/cm^2

Table S2. HIFU intensities and mechanical indices (MI) of 1.5 MHz transducer.

Acoustic power	Focal sound intensity (*I*)	Focal pressure	Mechanical index (MI)
0 W	0 W/cm^2	0 kPa	0
2 W	140 W/cm^2	2100 kPa	1.7
3 W	220 W/cm^2	2500 kPa	2.0
4 W	290 W/cm^2	2900 kPa	2.4
8 W	580 W/cm^2	4100 kPa	3.3
16 W	1200 W/cm^2	5900 kPa	4.7
32 W	2300 W/cm^2	8300 kPa	6.7

Table S3. Equations to calculate the M_n decreasing ratio and degradation rate constant k.[8] M_i is the initial number average molar mass (M_n) of the polymer, M_t is the number average molar mass of the sonicated sample at time t. M_o is the molar mass of the monomer unit.

Equation 1	M_n decreasing ratio $= \frac{M_i - M_t}{M_i}$
Equation 2	$\frac{1}{M_t} = \frac{1}{M_i} + k't$<br>$k' = \frac{k}{M_o}$

Table S4. M_n results of NCSPs with different conditions sonication.

Sonication		0 min	2 min	5 min	10 min	30 min	60 min
20 kHz	25% Amp (0.45 w cm^{-2})	108.5 (kDa)	102.4 (kDa)	98.7 (kDa)	86.3 (kDa)	74.4 (kDa)	63.3 (kDa)
	50% Amp (2.7 w cm^{-2})	108.5 (kDa)	98.1 (kDa)	86.9 (kDa)	72.3 (kDa)	49.3 (kDa)	33.3 (kDa)
	100% Amp (12.4 w cm^{-2})	108.5 (kDa)	73.7 (kDa)	58.4 (kDa)	47.2 (kDa)	32.7 (kDa)	32.4 (kDa)
1.5 MHz	32 W (2300 w cm^{-2})	108.5 (kDa)	100.2 (kDa)	51.7 (kDa)	40.6 (kDa)	33.3 (kDa)	32.4 (kDa)

Table S5. M_n results of FNCSPs with different acoustic power HIFU sonication.

1.5 MHz (30 min)	140 (w cm^{-2})	290 (w cm^{-2})	580 (w cm^{-2})	1200 (w cm^{-2})	2300 (w cm^{-2})
M_n	108.5 (kDa)	75.7 (kDa)	53.9 (kDa)	36.8 (kDa)	33.3 (kDa)

Table S6. M_n results of FNCSPs with HIFU sonication.

1.5 MHz (2300 w cm^{-2})	0 min	2 min	5 min	10 min
M_n	125.5 (kDa)	122.5 (kDa)	116.6 (kDa)	105.3 (kDa)

Table S7. Molar mass (M_n) changes and chain scission rate constant of star polymers and linear polymers with 20 kHz sonication.

Frequency	Polymers	Molecular weight (Mn)	Spanning molecular weight	Molecular weight Mn (60 min sonication)	Rate constant of chain scission (10^{-5} min^{-1})
20 kHz (12.4 W cm^{-2})	Star polymers (Mn 108.5 kDa)	108.5 kDa	53.5 kDa	32.4 kDa	10.8
	Linear polymers (Mn 47 kDa)	47 kDa	47 kDa	23.7 kDa	10.4

Table S8. Molar mass (M_n) changes and chain scission rate constant of star polymers and linear polymers with 1.5 MHz HIFU irradiation.

Frequency	Polymers	Molecular weight (Mn)	Spanning molecular weight	Molecular weight Mn (60 min sonication)	Rate constant of chain scission (10^{-5} min^{-1})
HIFU 1.5 MHz (2900 W cm^{-2})	Star polymers	108.5 kDa	53.5 kDa	32.4 kDa	10.8
	Linear polymers (Mn 47 kDa)	47 kDa	47 kDa	46.6 kDa	0.1

6. References

[1] G. R. Fulmer, A. J. M. Miller, N. H. Sherden, H. E. Gottlieb, A. Nudelman, B. M. Stoltz, J. E. Bercaw, K. I. Goldberg, *Organometallics* **2010**, *29*, 2176.

[2] M. Xuan, J. Fan, V. N. Khiêm, M. Zou, K. O. Brenske, A. Mourran, R. Vinokur, L. Zheng, M. Itskov, R. Göstl, A. Herrmann. *Adv. Mater.*, **2023**. *35*, 2305130.

[3] V. Hong, A. A. Kislukhin, M. G. Finn, *J. Am. Chem. Soc.* **2009**, *131*, 9986.

[4] A. G. Aioub, C. J. Higginson, M. G. Finn, *Org. Lett.* **2018**, *20*, 3233.

[5] S. Tekkam, M. G. Finn, *Org. Lett.* **2017**, *19*, 2833.

[6] Z. Shi, J. Wu, Q. Song, R. Göstl, A. Herrmann, *J. Am. Chem. Soc.* **2020**, *142*, 14725.

[7] (a) A. K. Pearce, A. B. Anane-Adjei, R. J. Cavanagh, P. F. Monteiro, T. M. Bennett, V. Taresco, P. A. Clarke, A. A. Ritchie, M. R. Alexander, A. M. Grabowska, C. Alexander, *Adv. Healthcare Mater.* **2020**, *9*, 2000892. (b) S. Pal, M. R. Hill, B. S. Sumerlin, *Polym. Chem.* **2015**, *6*, 7871; (c) M. Zou, P. Zhao, J. Fan, R. Göstl, A.

Herrmann, *J. Polym. Sci.*, **2022**, *60*, 1864-1870. (d) A. M. Striegel, *J. Biochem. Biophys. Methods*, **2003**, *56*, 117.

[8] M. J. Kryger, A. M. Munaretto, J. S. Moore, *J. Am. Chem. Soc.* **2011**, *133*, 18992.
